# Boosting Pseudocapacitive Behavior of Supercapattery Electrodes by Incorporating a Schottky Junction for Ultrahigh Energy Density

**DOI:** 10.1007/s40820-023-01016-6

**Published:** 2023-03-10

**Authors:** Selvaraj Seenivasan, Kyu In Shim, Chaesung Lim, Thangavel Kavinkumar, Amarnath T. Sivagurunathan, Jeong Woo Han, Do-Heyoung Kim

**Affiliations:** 1 School of Chemical Engineering, Chonnam National University, 77 Yongbong-Ro, Gwangju, 61186 Republic of Korea; 2 Division of Environmental Science and Engineering, Pohang University of Science and Technology (POSTECH), Pohang, 37673 Republic of Korea; 3 Department of Chemical Engineering, Pohang University of Science and Technology (POSTECH), Pohang, 37673 Republic of Korea

**Keywords:** Pseudo-capacitance, Negative electrode, Supercapattery, Atomic layer deposition, Energy density

## Abstract

Pseudo-capacitive negative electrodes remain a major bottleneck in the development of supercapacitor devices with high energy density because the electric double-layer capacitance of the negative electrodes does not match the pseudocapacitance of the corresponding positive electrodes. In the present study, a strategically improved Ni-Co-Mo sulfide is demonstrated to be a promising candidate for high energy density supercapattery devices due to its sustained pseudocapacitive charge storage mechanism. The pseudocapacitive behavior is enhanced when operating under a high current through the addition of a classical Schottky junction next to the electrode–electrolyte interface using atomic layer deposition. The Schottky junction accelerates and decelerates the diffusion of OH^‒^/K^+^ ions during the charging and discharging processes, respectively, to improve the pseudocapacitive behavior. The resulting pseudocapacitive negative electrodes exhibits a specific capacity of 2,114 C g^−1^ at 2 A g^−1^ matches almost that of the positive electrode’s 2,795 C g^−1^ at 3 A g^−1^. As a result, with the equivalent contribution from the positive and negative electrodes, an energy density of 236.1 Wh kg^−1^ is achieved at a power density of 921.9 W kg^−1^ with a total active mass of 15 mg cm^−2^. This strategy demonstrates the possibility of producing supercapacitors that adapt well to the supercapattery zone of a Ragone plot and that are equal to batteries in terms of energy density, thus, offering a route for further advances in electrochemical energy storage and conversion processes.

**Supplementary Information:**

The online version contains supplementary material available at 10.1007/s40820-023-01016-6.

## Highlights

- Incorporation of Schottky Junction increases the pseudocapacitive mechanism at higher current rate.
- The pseudocapacitance behavior of the positive and negative electrodes is balanced to construct a solid-state supercapattery device.
- An energy density of 236.14 Wh kg^−1^ is achieved for solid-state supercapattery device.

**Supplementary Information:**

The online version contains supplementary material available at 10.1007/s40820-023-01016-6.

## Graphical abstract

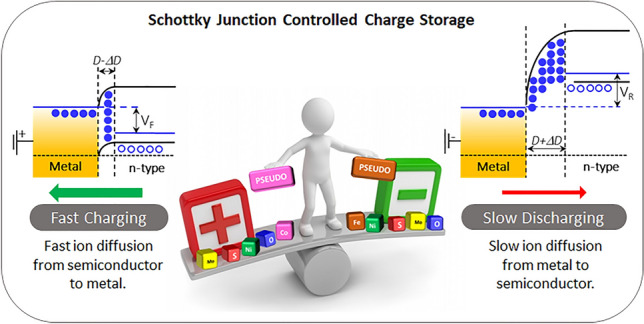

## Introduction

Supercapacitors and batteries are the primary candidates that have widespread usage and attract the most interest for further development [1]. Batteries have a larger retail market share owing to their capability of operating for a long duration with high energy density. For example, the global electric vehicles batteries market (GEVB) is expected to reach a valuation of USD 87.2 billion in the year 2027 [2]. Notably, supercapacitors push the boundaries by allowing the fusion of different electrodes as hybrid, asymmetrical and supercapattery devices, while corresponding metal anodes are mandatorily required for batteries (for example, Li, Na and Zn ion battery) [3–5]. In addition, supercapacitors provide attractive benefits such as a long cycle life, dependence on abundant elements, and compatibility with other applications [6].

A comparison of the classical electrical double layer (EDLC) and pseudocapacitive charge storage reveals that the latter is worth greater attention because it is analogous to a battery and thus presents broader opportunities for further development [7]. The rapid and reversible surface redox reaction in pseudocapacitive charge storage systems results in higher energy density and wider application prospects. In the pursuit of both high energy and power density, a new type of energy storage device configuration called supercapattery has emerged [8, 9]. In a conventional supercapattery configuration, EDLC and pseudocapacitive electrodes are combined to operate at a high energy density, although in a real-time application, each electrode must be capable of storing charge via both mechanisms to deliver a high energy output [10–12]. A classical method involves designing a high surface area electrode using an intrinsically pseudocapacitive material, whereby both the pseudocapacitive contribution (from the material) and EDLC contribution (owing to the high surface area) can be derived and summed-up as overall device capacity [13]. The inadequate specific capacity of negative electrodes to match positive electrodes is one of the remaining bottlenecks for the commercialization of supercapattery devices. The poor pseudocapacitive contribution of negative electrodes can limit the overall device capacity of the supercapattery device [14]. The research on pseudocapacitive negative electrodes is limited because of an inadequate choice of materials [15]. Exempting the numerous EDLC carbon materials such as carbon nanotubes, graphene, and reduced graphene oxide, few pseudocapacitive transition metal oxides/sulfides have been studied to match the specific capacity of positive electrodes [16, 17]. Therefore, designing compatible negative electrodes with sustained pseudocapacitive behavior is essential to realize the real-time application of a supercapattery device.

In this study, we designed positive and negative electrodes with a large surface area that had a large proportion of surface-active sites, as well as a Schottky junction next to the electrode–electrolyte interface through atomic layer deposition (ALD) to boost the pseudocapacitive behavior [18–20]. The presence of a Schottky junction controls the ion diffusion rate during the charging/discharging process and significantly improves the pseudocapacitive contribution to the overall specific capacity. Another unique advantage is that ALD-coated thin films are in the 10^–9^ g cm^−2^ range, so they do not increase the total mass, but provide the advantages of traditional core–shell structures resulting in high mass activity [21]. The aforementioned strategies proved successful as the manufactured supercapattery device demonstrated an energy density of 236.14 Wh kg^−1^, which fits well in the supercapattery zone of the Ragone diagram [22].

## Experimental Section

### Synthesis of the NiCo_2_S_4_/NiMo_2_S_4_/ALD-Co_3_O_4_ Positive Electrode

#### Synthesis of NiCo_2_S_4_ Nanoneedles

NiCo_2_O_4_ (NCO) nanoneedles were synthesized on Ni foam (NF) via a hydrothermal method. A piece of NF (2 × 5 cm^2^) was cleaned by ultrasonication using ethanol and deionized (DI) water for several minutes and then dried. The cleaned NF was transferred to a Teflon-lined autoclave containing a precursor solution prepared by dissolving 0.388 g of cobalt nitrate, 0.3 g of nickel nitrate, 0.3 g of urea, and 0.062 g of ammonium fluoride in 40 mL of DI water. The hydrothermal reaction was conducted at 120 °C for 6 h. Subsequently, the obtained NCO on NF was rinsed with DI water and dried at 60 °C. A wet sulfurization process was employed to drive the anion exchange reaction (AER) for converting NCO to NiCo_2_S_4_ (NCS). The sulfurization solution was prepared using 2 g of Na_2_S in DI water and the AER reaction was completed at 120 °C for 8 h. Finally, the obtained NCS on NF was rinsed with Ethanol and DI water, and then vacuum dried at 80 °C for further use.

#### Synthesis of NCS/NMS Hollow Cuboids

To synthesize NiCo_2_O_4_/NiMo_2_O_4_ (NCO/NMO), NiMo_2_O_4_ (NMO) cuboids were synthesized on NF/NCO following a hydrothermal method [23]. The precursor solution was prepared by dissolving 0.5 g of ammonium molybdate and 0.465 g of nickel nitrate in 40 mL of DI water. The hydrothermal reaction was conducted at 150 °C for 6 h. The wet sulfurization process was employed to drive the AER for converting NCO/NMO to NiCo_2_S_4_/NiMo_2_S_4_ (NCS/NMS). The AER duration was varied from 4 to 10 h to optimize the hollow structure formation. Finally, the obtained NCS/NMS on NF was rinsed with Ethanol and DI water, and then vacuum dried at 80 °C for further use.

#### Synthesis of NCS/NMS/ALD-Co_3_O_4_ Hollow Cuboids

Co_3_O_4_ (CoO) thin films were deposited using a homemade flow-type ALD reactor maintained at 175 °C and 800 mTorr. Cobalt cyclopentadienyl (Co(Cp)_2-_Sigma-Aldrich, USA) and ozone (5% in O_2_) were used as the cobalt precursor and counter reactant, respectively, in the ALD process [24]. Argon (99.999%) was used as both the carrier (50 sccm) and purging (250 sccm) gas. An ALD cycle comprised four steps, namely, a precursor pulse of 1.5 s, followed by precursor purge for 60 s, an ozone pulse of 2 s, and the final ozone purge for 60 s. The thickness of the CoO shell over NCS/NMS substrates was controlled by the number of ALD cycles (100, 200, and 300).

### Synthesis of the NMS/ALD-Fe_2_O_3_ Negative Electrode

#### Synthesis of NMS Hollow Cuboids

NMO cuboids were synthesized on cleaned bare NF, as described in the previous section. After rinsing and drying, the AER was conducted for 8 h. The obtained NMS on NF was rinsed with Ethanol and DI water, and then vacuum dried at 80 °C for further use.

#### Synthesis of NMS/FeO Hollow Cuboids

Fe_2_O_3_ (FeO) thin films were deposited through a homemade flow-type ALD reactor maintained at 175 °C and 800 mTorr [25]. Bis(bis(trimethylsilyl)amide) iron(II) (Fe(btmsa)_2_—Hansol Chemicals, South Korea) and ozone (5% in O_2_) were used as the iron precursor and counter reactant, respectively, in the ALD process. An ALD cycle comprised four steps, i.e., a precursor pulse of 1.5 s, followed by precursor purge for 60 s, an ozone pulse of 2 s, and final ozone purge for 60 s. The thickness of the FeO shell over NMS substrates was controlled by the number of ALD cycles (150, 300 and 450).

### Characterization

The crystallinity of each sample was analyzed using a high-resolution X-ray diffractometer (XRD; PANalytical) using a 3D-PIXcel detector and equipped with Cu–Kα radiation at 60 kV and 55 mA. High-resolution X-ray photoelectron spectroscopy (XPS) was employed with Kα radiation and seven Channeltron detectors. High-resolution scanning electron microscopy (HR-SEM; JEOL JSM-7500F) coupled with an energy-dispersive X-ray spectroscopy (EDS) analyzer with 15 kV acceleration and high-resolution transmission electron microscopy (HR-TEM, TECNAI G2 F20) were used to study the structural and morphological properties of the electrodes.

### Electrochemical Measurements

The electrochemical measurements were conducted in a three-electrode system containing 2 M KOH electrolyte, wherein a saturated calomel electrode (SCE) and Pt foil were used as reference and counter electrodes, respectively. A WonATech WBCS3000 automatic battery cycler was used for the electrochemical experiments. Electrochemical impedance spectroscopy (EIS) measurements were conducted at frequencies ranging from 10^–2^ to 10^6^ Hz with an amplitude of 10 mV. The electrochemically active surface area (ECSA) was estimated by calculating the electric double layer capacitance (*C*_dl_ = d*(∆I)/*d*ν*) in the non-Faradaic potential region. Cyclic stability tests were conducted at a constant charging/discharging current density with frequent replacement of the electrolyte. The specific capacitance, *C*_s_ (F g^−1^), and specific capacity, *C* (C g^−1^), of the electrodes were calculated from the galvanostatic charge/discharge (GCD) curves using the following formulae:

1 $${C}_{s}=\frac{2 I\int V\mathrm{d}t}{m {\Delta V}^{2}}$$

2 $$C=\frac{2 I\int V\mathrm{d}t}{m \Delta V}$$

where *I*(A) is the discharge current, *ΔV*(V) is the potential range, and *m*(g) is the mass of the active materials.

### NCS/NMS/CoO||NMS/FeO Device

For the fabrication of the supercapattery device, NCS/NMS/CoO and NMS/FeO electrodes were employed as positive and negative electrodes, respectively, with 2 M KOH/PVA gel electrolyte. For the constructed energy storage device, the mass ratio between the two electrodes was calculated according to the following formula [24]:

3 $$\frac{{m}^{+}}{{m}^{-}}=\frac{{C}^{-}\Delta {V}^{-}}{{C}^{+}\Delta {V}^{+}}$$

where *C*^*−*^ and *C*^+^ are the specific capacities, and *m*^*−*^ and *m*^+^ are the masses of the negative and positive electrodes, respectively. *ΔV*^*−*^ and *ΔV*^+^ denote the voltage window of the two electrodes, respectively. The energy density, *E* (Wh kg^−1^), and power density, *P* (W kg^−1^), of the supercapattery device were calculated as follows:

4 $$E= \frac{I\int V dt }{3.6}$$

5 $$P= \frac{3600 E}{\Delta t}$$

where *Δt*(s) is the discharge time and *I*(A g^−1^) is the discharge current density.

### Computational Details

All density functional theory (DFT) calculations were performed using the Vienna ab Initio Simulation Package (VASP) [26]. The Perdew–Burke–Ernzerhof (PBE) functional of the generalized gradient approximation (GGA) was used for the exchange–correlation energies [27, 28]. The energy cut-off for the plane-wave basis set was set at 400 eV, and the Brillouin-zone was sampled using DFT Monkhorst–Pack 5 × 5 × 5, 2 × 2 × 1, and 4 × 4 × 1 k-point meshes for the bulk, surface, and charge analysis models, respectively [29]. For the structural optimization, all atoms were relaxed using a conjugate-gradient algorithm until the difference in the total force was < 0.03 Ev Å^−1^ with a convergence criterion of 10^–4^ eV for the total energy, while spin polarization was also taken into considerations [30].

For all slab models, termination with a lower surface energy was selected. Equation (6) was used to calculate the surface energy ($\upgamma$):

6 $$\upgamma =\frac{{\mathrm{E}}_{\mathrm{slab}}-n{E}_{\mathrm{bulk}}}{2A}$$

where ${E}_{\mathrm{slab}}$ is the total energy of the slab model, n is the stoichiometry parameter, ${E}_{bulk}$ is the total energy of the bulk model, and A is the surface area of the slab model. Additionally, vacuum layer spacing of ~ 15 Å was employed.

In this study, three hybrid electrode models were constructed: 1) NCS/NMS 2) NCS/NMS/CoO/OH^–^ and 3) NMS/FeO/K^+^. For the NCS/NMS model, NCS(110) was selected because its surface energy is lower than that of NCS(100) and NCS(111), while NMS(110) was used because the hydrothermal method was used for NCS/NMS synthesis, during which Co atoms were hydrothermally replaced with Mo atoms. To ensure a suitable comparison with NCS/NMS/CoO/OH^–^, NMS(110) was adopted for the NMS/FeO/K^+^ model. Moreover, eight layers for the NCS/NMS surface model were used while bottom four layers were fixed, so were for NCS/NMS/CoO/OH^–^ model. Similarly, eight layers were used with bottom four layers fixed for NMS/FeO/K^+^.

For NCS/NMS/CoO/OH^–^ and NMS/FeO/K^+^, Co_3_O_4_(110) and Fe_2_O_3_(0001) were selected as the most stable facets for electrode purposes according to literature [31, 32]. The VESTA package was used for structural visualization [33].

## Results and Discussion

### Physical Characterizations of NCS/NMS/CoO Positive Electrode

**Fig. 1:**
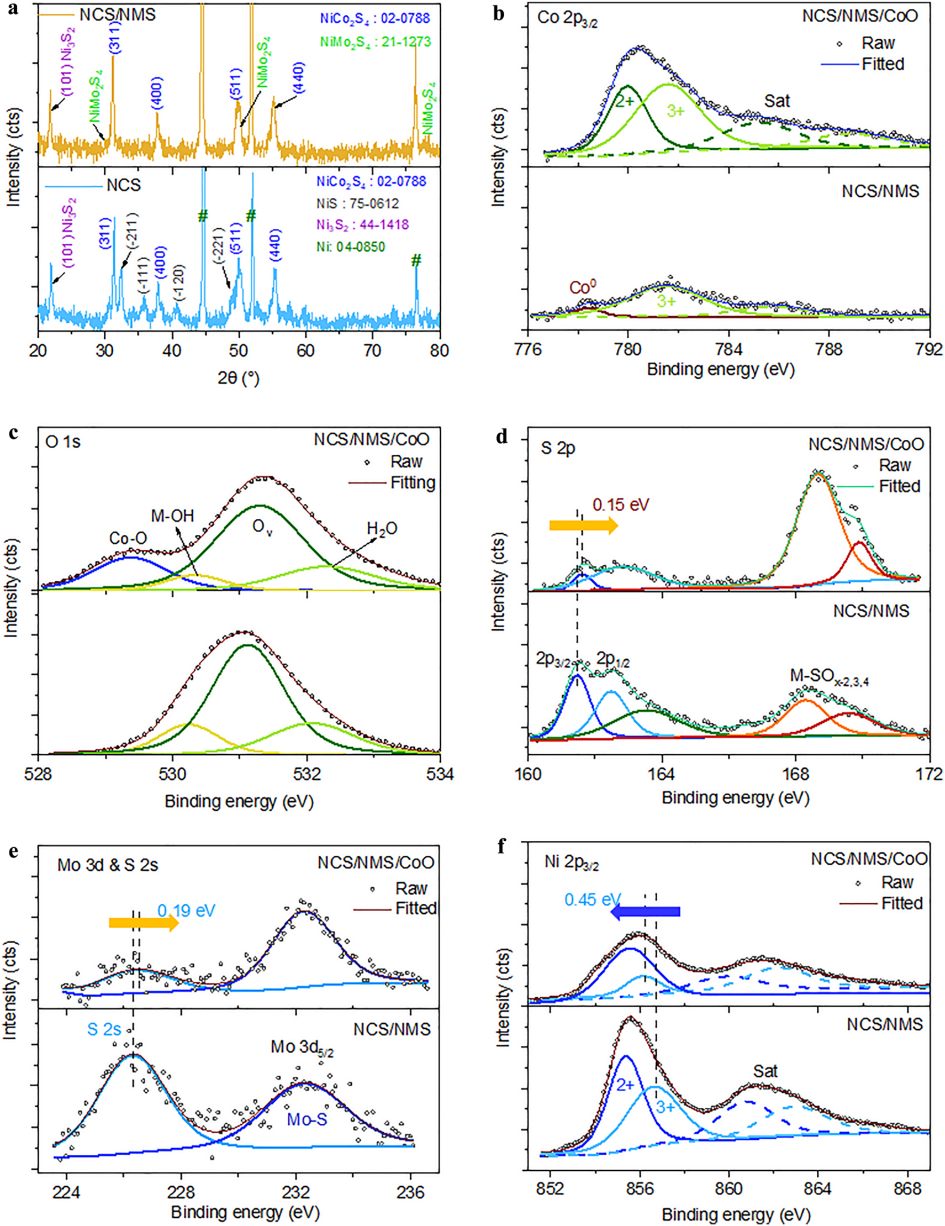
XRD patterns of **a** NCS/NMS (yellow) and NCS (blue) positive electrodes. Comparison of high-resolution XPS profiles, **b** Co 2*p*, **c** O 1*s*, **d** S 2*p*, **e** Mo 3*d*, and **f** Ni 2*p* of NCS/NMS, and NCS/NMS/CoO electrodes

Figure 1a shows the XRD spectra of the prepared positive electrodes after the AER. The as-prepared NCS showed distinct peaks of thiospinel NiCo_2_S_4_ stoichiometry (JCPDS No. 02-0788) at 31.35°, 38.04°, 49.86°, and 55.41°, which correspond to the (311), (400), (511), and (440) planes of the NCS crystal. Additional peaks of Ni_3_S_2_ (JCPDS No. 44-1418) and NiS (JCPDS No. 75-0612) stoichiometry were also observed. All samples showed the diffraction peaks of Ni foam (JCPDS: 04-0850) in addition to the peaks of the coated material. The NCS/NMS structure showed a few more pronounced peaks of NiMo_2_S_4_ at 50.15° and 77.75° along with the inherent peaks of NCS [34]. This observation suggested that the NCS/NMS structure has separate crystals of NCS and NMS, and does not correspond to the Mo-doped stoichiometry of NCS.

The surface compositions of bare and ALD-CoO-coated NCS/NMS electrodes were analyzed by XPS. The survey spectra of all samples are shown in Fig. S1a, b. The high-resolution Co 2*p* spectrum of NCS/NMS revealed Co^3+^ (781.61 eV) as the major chemical state corresponding to the NCS phase with a small amount of elemental Co^0^ (778.37 eV) that represented the existence of oxygen vacancies (Fig. 1b) [35]. The incorporation of CoO thin layers induced the Co^2+^ (779.97 eV) state and the overall Co 2*p* peak intensities increased dramatically owing to the enrichment of the surface with Co atoms. However, the major 3 + state revealed the Co_3_O_4_ spinel structure with Co^2+^ ions in tetrahedral interstices and Co^3+^ ions in the octahedral interstices of the cubic close-packed lattice of oxide anions. In addition, the disappearance of the elemental Co^0^ peak indicated the oxidation by exposure to reactive ozone molecules in the ALD process. As shown in Fig. 1c, both electrodes show two major peaks at 531.12 and 530.40 eV corresponds to oxygen vacancies and M‒OH bond, respectively [36]. The Co‒O peak (529.51 eV) emerged after the deposition of an ultrathin ALD layer. Figure 1d shows the deconvolution of S 2*p* peaks with doublet peaks at 162.51 and 161.48 eV corresponding to the S_2_^2−^ (disulfide) and S^2−^ (sulfide) ligands, respectively [37]. The M‒SO_x_ (metal sulfate/metal sulfite) peaks observed in the range of 165.00–170.00 eV originated from surface oxidation by air. The high-resolution Mo 3*d* spectrum showed a single peak at 232.26 eV corresponding to Mo 3*d*_5/2_ and this peak was attributed to the + 3 oxidation states of Mo atoms (Fig. 1e) [34]. The high-resolution Ni 2*p* spectrum of NCS/NMS clearly showed the dominant Ni^2+^ (855.35 eV) and Ni^3+^ (856.72 eV) states of mixed NCS/NMS stoichiometry (Fig. 1f). Upon incorporation of ALD-CoO, NiO was formed beneath the CoO thin layer, as evidenced by the dominant Ni^2+^ (855.54 eV) peak in the NCS/NMS/CoO sample [21]. The positive peak shift of 0.15 and 0.19 eV in the S 2*p* and S 2*s* spectra, respectively, originated from the low electron density near the S atoms after the incorporation of the CoO thin layer. The Ni 2*p* spectrum showed a relatively negative shift of 0.45 eV owing to the high electron density around the Ni atoms in the NMS phase. The electronic equilibrium attained by the sharing of the outer shell electrons between the NMS phase and CoO thin layer further generated high local electronic interactions, and this presented a freeway for electron transfer. The diminished S 2*p*, Ni 2*p*, Mo 3*d*, and S 2*s* peaks observed after ALD-CoO incorporation indicated that the metal-sulfide structure was wrapped by the conformal CoO coating.

**Fig. 2:**
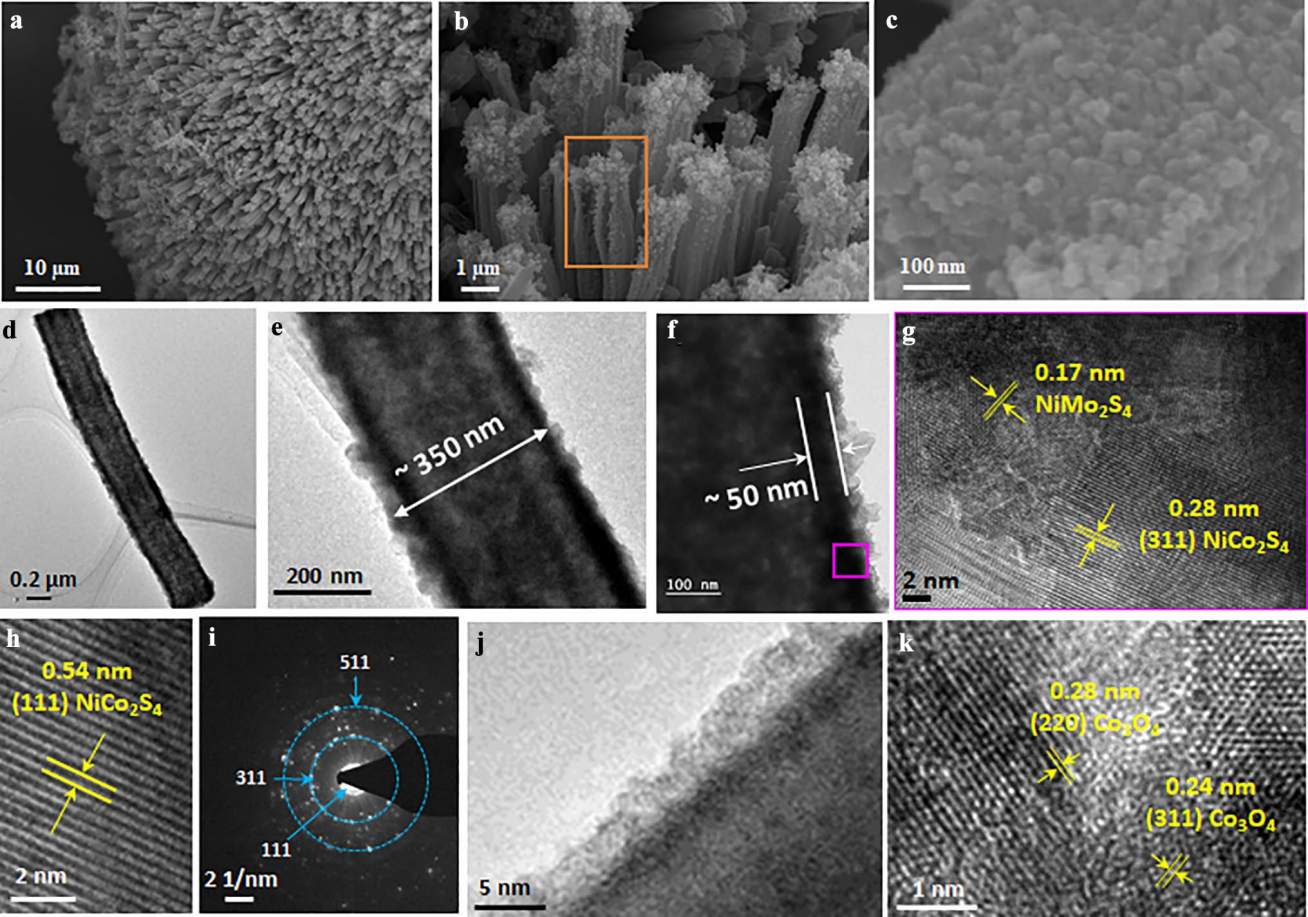
**a–c** HR-SEM images of bare NCS/NMS electrode. HR-TEM images of **d–i** bare NCS/NMS electrode with diffraction plane analysis and SAED pattern and **j** NCS/NMS/CoO (200 cycles), and **k** NCS/NMS/CoO (300 cycles)

Figure S2 shows that the as-prepared NCS electrodes had a well-defined flower structure with multiple nano-needles connected to single root. Figure 2a shows that the as-prepared NCS/NMS electrodes had cuboidal structures with cuboidal walls composed of several NCS and NMS nanoparticles. Especially, a broken nano-cuboid in Fig. 2b illustrates the hollow features of the formed NCS/NMS cuboids. The hollow structures were formed by the wet sulfurization process and have also been observed in various metal-oxide to metal-sulfide transformations[38]. Hollow structures are well known for their large surface area, electrolyte contact, and fast charge transfer kinetics. The HR-TEM images in Fig. 2d–f show the hollow nature of the NCS/NMS cuboids, with cuboid and wall thicknesses of ~ 350 and ~ 50 nm, respectively. The image of a wall portion at higher magnification is shown in Fig. 2g, h; it shows NCS crystals with *d*-spacings of 0.28 and 0.54 nm that correspond to the (311) and (111) planes [39]. In addition, NMS crystals with 0.17 nm lattice *d*-spacing were observed [40]. The selected area electron diffraction (SAED) pattern observed for the cuboidal wall reveals the polycrystalline nature of NCS through the formation of concentric circles with bright spots. The major peaks observed by XRD analysis, including the (311), (400), and (511) planes, were indexed in the SAED pattern. Figure 2j shows the conformal coating of the CoO thin layer over the cuboid with a uniform thickness of ~ 3.25 nm (200 ALD cycles). An image of the CoO thin layer at a higher magnification is presented in Fig. 2k, showing Co_3_O_4_ crystals with *d*-spacings of 0.24 and 0.28 nm corresponding to the (311) and (220) planes, respectively [41]. Furthermore, the scanning transmission electron microscopy energy-dispersive spectroscopic (STEM-EDS) images in Fig. S3 reveal the homogeneous distribution of Co, Ni, Mo, S and O elements. These results confirm the formation of the hollow NCS/NMS/CoO core–shell structure with excellent interface properties.

### Physical Characterizations of NMS/FeO Negative Electrode

As illustrated in Fig. S4, the XRD spectrum of the NMO negative electrode showed low-intensity peaks of the NMO phase (JCPDS No. 16-0291) with additional peaks of MoO_3_ (JCPDS No. 01-0706) owing to a lack of crystallinity. The XRD spectrum of the NMS electrode showed clear peaks of the NMS phase (JCPDS No. 21-1273) at 29.64°, 50.45°, and 78.06°. Ni foam exposed to the Na_2_S solution during AER and led to the formation of Ni_3_S_2_ phase. The surface composition of the NMS negative electrode was analyzed by XPS, and the results are shown in Fig. S5. The high-resolution Ni 2*p* spectrum of NMS clearly shows the dominant Ni^2+^ (855.48 eV) state and Ni^3+^ (856.94 eV) state of NMS stoichiometry (Fig. S5a) [42]. The high-resolution Mo 3*d* spectrum shows a single peak at 232.26 eV that corresponds to Mo 3*d*_5/2_ and can be attributed to the + 3 oxidation states of Mo atoms (Fig. S5b). The emergence of S 2*s* peak at 226.66 eV confirmed the successful conversion of NMO to NMS. Figure S5c shows the deconvolution of S 2*p* peaks into doublet peaks at 162.79 and 161.77 eV corresponding to the S_2_^2−^ (disulfide) and S^2−^ ligands, respectively [37]. As shown in Fig. S5d, the oxygen vacancy peak at 531.22 eV was the major peak representing one of the surface-active sites of the NMS negative electrode. The Fe 2*p* spectrum (Fig. S5e) exhibits two significant peaks centered at 712.15 and 725.65 eV, which correspond to 2*p*_3/2_ and 2*p*_1/2_ spin orbitals, respectively.

**Fig. 3:**
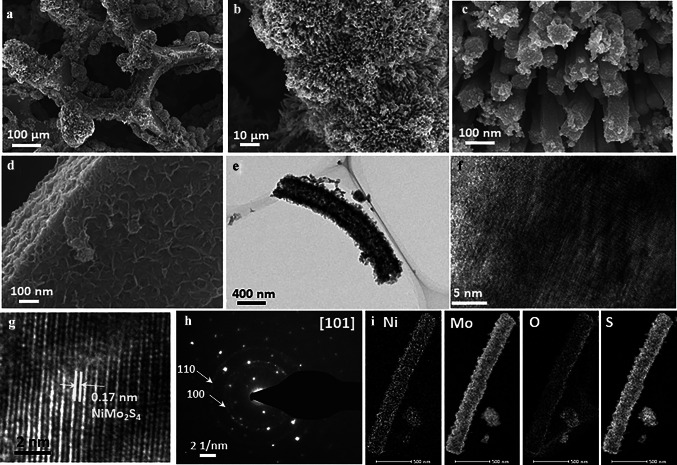
**a–d** HR-SEM images of the bare NMS electrode. **e–h** HR-TEM images of the bare NMS electrode with diffraction plane analysis and SAED pattern. **i** Elemental mapping of the NMS electrode

The HR-SEM images of the bare NMS negative electrodes are shown in Fig. 3a–d. From Identical hollow cuboid structures were formed after the wet sulfurization process with a thick wall composed of NMS nanosheets as shown in Fig. 3e, f. At high magnification, clear differences could be seen between Figs. 2c and 3d. While the NCS/NMS cuboids mainly composed of nanoparticles, the NMS electrodes were made up of nanosheets. This difference is because of the formation of a unique S‒Mo‒S layered structure linked through van der Waals forces [43]. The NMS crystal lattice *d*-spacing was 0.17 nm (Fig. 3g). The well-ordered bright spots arranged in a straight line observed in the SAED pattern corresponded to the [101] facet and indicated that the NMS was highly crystalline (Fig. 3h). The perfect circles were indexed to the (100) and (110) planes of the NMS crystals. Collectively, these results show that the NMS cuboids had both polycrystalline and single crystalline properties. The STEM-EDS images in Fig. 3i demonstrate the homogeneous distribution of Mo, Ni, O, and S elements.

### Electrochemical Performance of the NCS/NMS/CoO Positive Electrode

**Fig. 4:**
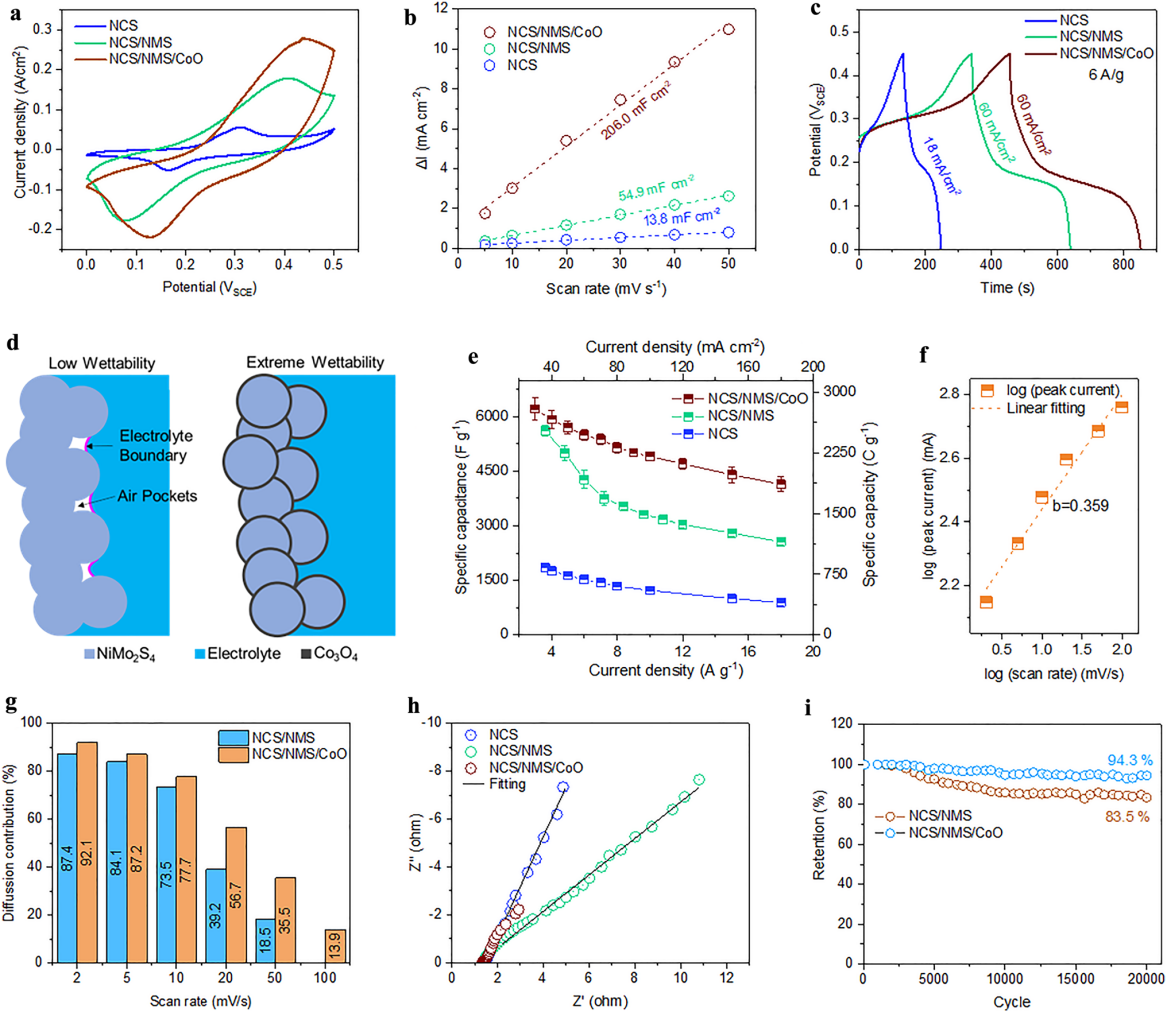
**a** CV curves of NCS, NCS/NMS, and NCS/NMS/CoO electrodes at 5 mV s^−1^ scan rate. **b** Estimation of *C*_dl_. **c** GCD curves of NCS, NCS/NMS, and NCS/NMS/CoO electrodes at 6 A/g current density. **d** Schematic of improved wettability by CoO ALD layer. **e** Comparison of the specific capacities of NCS, NCS/NMS, and NCS/NMS/CoO electrodes. **f** Plot of logarithmic scan rate versus logarithmic peak current. **g** Calculated contribution of diffusion-controlled charge storage mechanism for NCS/NMS and NCS/NMS/CoO electrodes. **h** Nyquist plot at 0.25 V and **i** plot of capacity retention

Figure S6 shows the CV plots of the NCS, NCS/NMS and NCS/NMS/CoO electrodes at various scan rates in 2 M KOH electrolyte. OH^–^ is the anion with the highest ionic conductivity and the highest mobility in water, while K^+^ is the cation with the second-highest ionic conductivity after H_3_O^+^. Furthermore, the smaller size of the OH^–^ anions increased the likelihood of intercalation, thus improving the pseudocapacitive behavior. Figure 4a shows the comparative CV curves of NCS, NCS/NMS, and NCS/NMS/CoO electrodes between 0 and 0.5 V at 5 mV s^−1^. All potentials are mentioned relative to the SCE unless specified otherwise. The NCS electrode showed oxidation and reduction peaks at 0.310 and 0.164 V, respectively, corresponding to the inherent pseudocapacitive behavior of NiCo_2_S_4_ [44]. After the formation of the NCS/NMS structure, the oxidation peaks shifted positively (0.177 V) and the reduction peak shifted negatively (− 0.176 V) owing to the incorporation of the additional Mo^4+^/Mo^6+^ redox couple in the electrode. The electrochemical redox process involves the following reactions [45]:

$${\text{M - S }} + {\text{ OH}}^{ - } \leftrightarrow {\text{ M}} - {\text{SOH }} + {\text{ne}}^{ - } \left( {{\text{M}} = {\text{Ni}}^{2 + /3 + } ,{\text{ Co}}^{2 + /3 + } ,{\text{ and Mo}}^{4 + /6 + } } \right),$$

$$\begin{aligned} & {\text{Co}}\left( {{\text{OH}}} \right)_{2} + {\text{ OH}}^{ - } \leftrightarrow {\text{ CoOOH }} + {\text{H}}_{2} {\text{O }} + {\text{ e}}^{ - } \left( {{\text{Co}}^{2 + } \;{\text{and}}\;{\text{Co}}^{3 + } } \right),{\text{ and}} \\ & {\text{CoOOH }} + {\text{ OH}}^{ - } \leftrightarrow {\text{ CoO}}_{2} + {\text{ H}}_{2} {\text{O }} + {\text{ e}}^{ - } \left( {{\text{Co}}^{3 + } \;{\text{and Co}}^{4 + } } \right) \\ \end{aligned}$$

The opposite shift in redox peaks compared to the bare NCS/NMS electrode indicated the additional contribution of Co^2+^/Co^3+^ Co^3+^/Co^4+^ redox couples in the pseudocapacitive reaction. The clear difference in the capacitive areas of the curves of NCS/NMS and NCS electrode illustrated the synergic effect of mixed metal sulfides (M‒S) present in the NCS/NMS electrode and the large surface area obtained by the hollow structure. The *C*_dl_ values of each electrode were obtained using the non-faradaic CV curves shown in Fig. S7. The *C*_dl_ of the NCS/NMS electrode was measured to be 54.9 mF cm^−2^, which is approximately 4.5 times higher than that of the NCS electrode (Fig. 4b). The capacitive areas of the NCS/NMS electrode curves were further extended with the incorporation of thin CoO films due to the ~ 3.75-fold increase in ECSA. The contact angle (*θ*) measurement shown in Fig. S8, shows significant reduction in contact angel before and after the CoO deposition. The CoO incorporation improved the wettability of NCS/NMS electrodes by exposing the electrolyte inaccessible areas to the electrolyte as shown in Fig. 4d. Owing to the ultra-high vacuum (UHV) processing conditions, the metal precursors could reach the trenches of the electrode surface that could not be physically reached by the electrolyte which increases the hydrophilicity of the electrode surface and results in an exponential increase in electrode–electrolyte interactions [46].

The GCD curves were obtained in the 0–0.45 V range to avoid polarization in aqueous electrolyte. The GCD curves in Fig. 4c for all electrodes exhibit a nonlinear plateau structure, indicating pseudocapacitive electrochemical behavior [47]. The NCS/NMS/CoO electrode had the longest discharge time, which indicated its superior energy storage capability. Figure 4e shows the specific capacity of the NCS/NMS/CoO (10 mg cm^−2^), NCS/NMS (10 mg/cm^2^), and NCS (3 mg cm^−2^) electrodes based on the GCD curves at current densities ranging from 3 to 18 A g^−1^. When calculating the specific capacity/capacitance of the active material, the mass of the current collector was not considered because the NF was completely covered by the active material. This means that the contribution of the NF to the charge storage was negligible. The obtained specific capacity was thus exclusively derived from the active material–electrolyte interface. Specific capacities of 2794.5, 2530.2, and 831.8 C g^−1^ were achieved for NCS/NMS/CoO, NCS/NMS, and NCS electrodes at 3.0 A g^−1^, respectively. In terms of specific capacitance, the NCS/NMS/CoO electrode achieved 6210.0 F g^−1^ at 3.0 A g^−1^. These values are a new benchmark for all types of transition metal-based positive electrodes for charge storage application (Table S1). The discharge time gradually decreased with increasing current density because of inadequate time for electrolyte ion diffusion, ultimately resulting in a lower specific capacity (Fig. S9). However, the incorporation of CoO significantly improved the rate capability of NCS/NMS might originate from the improved pseudocapacitive contribution to the overall specific capacity at the high current rate. To verify this, we have calculated the induvial contribution of EDLC and pseudocapacitive mechanism to the overall specific capacity with and without the CoO shell layer. By assuming the semi-infinite linear diffusion, the capacitive contribution (*C*_EDLC_) can be calculated using the Trasatti method (Fig. S10) [48, 49]. The calculated diffusion contribution (*C*_D_) to the total specific capacity (*C*_T_) before and after the incorporation of CoO ALD layer was plotted against the scan rate (Fig. 4g). At a slow scan rate, the diffusion-controlled ion intercalation is dominant because sufficient time is available to execute the slow ion migration and intercalation. The EDLC clearly becomes dominant at higher scan rates where only ion adsorption on the surface can happen with a large current rate. The CoO thin layer significantly increases the diffusion process at all scan rates compared to the bare NCS/NMS electrode. To verify the obtained values, the power-law equation was employed:

7 $$i=a{v}^{b}$$

8 $$\mathrm{log}i= \mathrm{log}a+b \mathrm{log}v$$

where *i* is the redox peak current (mA), *v* is the scan rate (mV/s), and *a* and *b* are constants. The value of slope (*b*) has three well defined regions: 0.5 ≥ *b* refers to the diffusion-controlled region, 1 > *b* ≥ 0.5 refers to the transition region, and *b* ≥ 1 refers to the capacitive region [50, 51]. The plot of log *v* versus log *i* (reduction peak from Fig. S6c) is given in Fig. 4f. The slope was determined as *b* = 0.359, which confirmed the dominance of the diffusion-controlled pseudocapacitive mechanism throughout the potential window. This indicates significant reversibility and high ion diffusion rate during a rapid high current rate electrochemical redox reaction.

The Nyquist plots for the NCS, NCS/NMS, and NCS/NMS/CoO electrodes are shown in Fig. 4h. The equivalent circuit used for impedance data fitting is shown in Fig. S11a and the fitted results are listed in Table S2. The low *R*_s_ value of the NCS/NMS/CoO electrode compared to the pristine electrode due to electronic interaction achieved between NCS/NMS‒CoO interfaces. The plots show that the NCS/NMS/CoO electrode had the lowest *R*_CT_ value compared to all other electrodes, indicating that the ultra-thin CoO greatly improved charge tunneling at the interface. The cyclic stability of bare and CoO-coated NCS/NMS electrode was tested at 10.8 A g^−1^ for 20,000 charge/discharge cycles in 2 M KOH electrolyte. Only 5.7% capacity loss was observed for the NCS/NMS/CoO electrode, whereas NCS/NMS lost 16.5% of its initial specific capacity, as shown in Fig. 4i. This suggests that the CoO layer preserved the structural integrity of the NCS/NMS electrode throughout the charging/discharging process. The insignificant change in morphology after 20,000 cycles demonstrates the excellent durability of the NCS/NMS/CoO nanostructures and their resistance to volume expansion or agglomeration phenomena during the charge/discharge processes (Fig. S12a, b). The disappearance of the M‒O peak at 529.56 eV and advent of the M‒OH peak at 530.16 eV indicated the conversion metal oxide to corrosion resistive metal hydroxides and (oxy)hydroxides (Fig. S12c) [21]. The S 2*p* spectrum showed slightly weaker M‒SO_x_ peaks, indicating the formation of M‒OOH phase (Fig. S12d).

### Electrochemical Performance of the NMS/FeO Negative Electrode

**Fig. 5:**
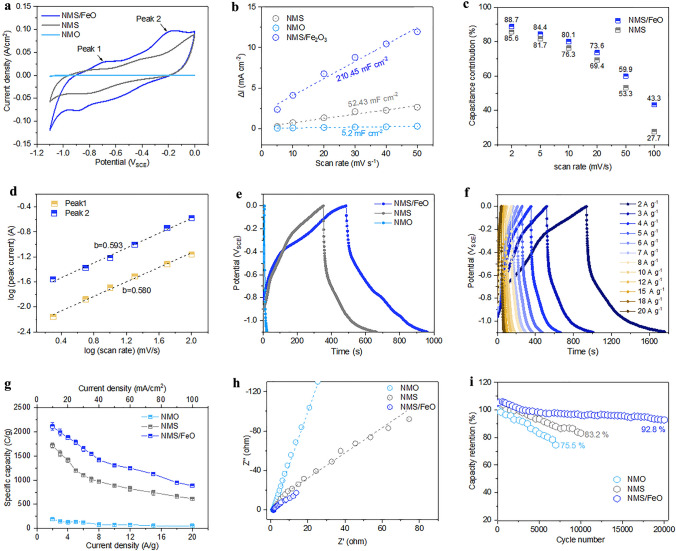
**a** CV curves of NMO, NMS, and NMS/FeO electrodes at 5 mV s^−1^ scan rate. **b** Estimation of *C*_dl_. **c** Calculated contribution of diffusion-controlled and EDLC charge storage mechanism for the NMS and NMS/FeO electrodes. **d** Plot of logarithmic scan rate versus logarithmic peak current. **e** GCD curves at 3 A g^−1^ current density. **f** GCD curve of NMS/FeO electrode at various current density. **g** Comparison of the specific capacities of NMO, NMS, and NMS/FeO electrodes. **h** Nyquist plot at ‒0.50 V and **i** Capacity retention

The CV curves of NMO, NMS, and NMS/FeO negative electrodes between − 1.1 and 0.0 V at various scan rates are given in Fig. S6e–h. Figure 5a shows the comparative CV curves of NMO, NMS, and NMS/FeO electrodes at 5 mV s^−1^ scan rate. The shape of the CV curves represents the surface redox-mediated pseudocapacitive charge storage mechanism with a few layers of the active material at the electrode‒electrolyte interface participating in the redox reaction [47]. The NCS phase was intentionally removed (from negative electrode) to obtain a nanosheet like morphology to boost the pseudocapacitive mechanism in the negative potential window. The unique structures of S‒Mo‒S nanosheets can provide sufficient space between the sheets for K^+^ ion intercalation/de-intercalation [52]. The NMS electrode shows two strong symmetrical redox peaks at − 0.788 and − 0.408 V corresponding to the Mo^4+^/Mo^6+^ and Ni^2+^/Ni^3+^ redox couples present in the electrode [53]. The peak current and peak intensity of the NMS electrode increased with the increasing scan rate and without any deformation of shape resulted superior rate capability. Specifically, the boosted electrochemical performance of the NMS/FeO heterostructure could be attributed to the additional Fe–O active sites (Fe^2+^/Fe^3+^ and Fe^3+^/Fe^4+^ redox couples) and typical pseudo-capacitance behavior of ALD-Fe_2_O_3_ by the following redox reaction [16]:

$${\text{Fe}}_{2} {\text{O}}_{3} + \, 2{\text{e}}^{ - } + \, 3{\text{H}}_{2} {\text{O }} \leftrightarrow {\text{ Fe}}\left( {{\text{OH}}} \right)_{2} + \, 2{\text{OH}}^{ - } \left( {{\text{Fe}}^{2 + } \;{\text{and}}\;{\text{Fe}}^{3 + } } \right)$$

The comparative integrated area of NMS compared to NMO shows the superior charge storage capability of NMS, due to the increased surface area of NMS hollow cuboids and the pseudocapacitive nature of charge storage. The measured *C*_dl_ of NMS (52.4 mF cm^−2^) was approximately one order larger than that of NMO (5.2 mF cm^−2^), revealing that the AER induced larger microroughness (Fig. 5b). The advantages of the thin FeO layer are identical to CoO on NCS/NMS positive electrodes. The estimated pseudocapacitive contribution was 88.7% and 43.28% at 2 and 100 mV s^−1^, respectively (Figs. 5c and S10). The plot of log *v* versus log *i* (peak 1 and peak 2 from Fig. 5a) is given in Fig. 5d. The slope was determined for peak 1 and peak 2 as *b*_*1*_ = 0.593 and *b*_*2*_ = 0.580, respectively. Both values are at the beginning of the transition mode, which confirms the dominance of the pseudocapacitive mechanism throughout the potential window [47].

Figure 5e depicts the GCD curves of the negative electrodes at 3 A g^−1^ in the potential range from ‒1.1 to 0.0 V. Notably, the triangle shape of the NMO GCD curve showed pure EDLC behavior because of the lack of surface-active sites. The analogous time of charging and discharging with a symmetric profile implied high columbic efficiency of the NMS and NMS/FeO electrodes. The GCD plot of NMS/FeO obtained at various current densities is shown in Fig. 5f. Figure 5g shows the specific capacity of the NMO (5 mg cm^−2^), NMS (5 mg cm^−2^), and NMS/FeO (5 mg cm^−2^) electrodes based on the GCD curves at current densities ranging from 2 to 20 A g^−1^. The NMS/FeO electrode achieved a specific capacity of 2,114 C g^−1^ at 2 A g^−1^, whereas the NMS and NMO electrodes reached specific capacities of 1,720 and 189 C g^−1^, respectively. The obtained values are a new benchmark compared to both carbon- and inorganic-based negative electrode materials reported for supercapacitors (Table S3).

Figure 5h shows the Nyquist plot at an applied potential of ‒ 0.5 V, which is fitted with the equivalent circuit shown in Fig. S11a. The fitted results are given in Table S2. The smaller series resistance (*R*_s_) and *R*_CT_ of the NMS/FeO electrode as compared to those of other negative electrodes reveal the improvement in overall conductivity and interface kinetics, respectively. The K^+^ ion diffusivity was calculated using the Warburg region by plotting the inverse square root of angular frequency (*ω*^*−1/2*^) and real impedance (*Z´*) as shown in Fig. S13. The ionic coefficients of the NMS and NMS/FeO electrodes were thus determined to be 0.395 × 10^–8^ and 1.259 × 10^–8^ cm^2^ s^−1^, respectively [54, 55]. It shows that the FeO shell layer provides more diffusion path for K^+^ ions.

The long-term cycling stability of the electrodes was evaluated after 20,000 GCD cycles at a current density of 10 A g^−1^. The NMS/FeO electrode displayed 93.8% of its original specific capacity after 20,000 cycles. The bare NMS electrode retained 83.2% of the specific capacity after 10,000 cycles (Fig. 5i). The thin layer of FeO minimized the deterioration of NMS in the alkaline electrolyte. The observed insignificant change in morphology indicates the excellent durability of the NMS/FeO nanostructures and their resistance to volume expansion or agglomeration phenomena during the charge/discharge processes (Fig. S14). The S 2*p* spectrum showed slightly weak 2*p*_3/2_ and 2*p*_1/2_ peaks and high intensity M‒SO_x_ peaks, indicating the formation of M‒OOH phase like NCS/NMS/CoO electrodes (Fig. S2c). The appearance of the M-OH peak at 530.52 eV indicated the conversion of M‒S to M‒OH, Fig. S2d. However, the significant S 2*s* and S 2*p* peaks indicate that Ni‒S and S‒Mo‒S active sites are available in the electrolyte permeable region near the surface after 20,000 charge/discharge cycles.

### Analysis of Enhanced Pseudo Capacitance Under a High Current Rate

**Fig. 6:**
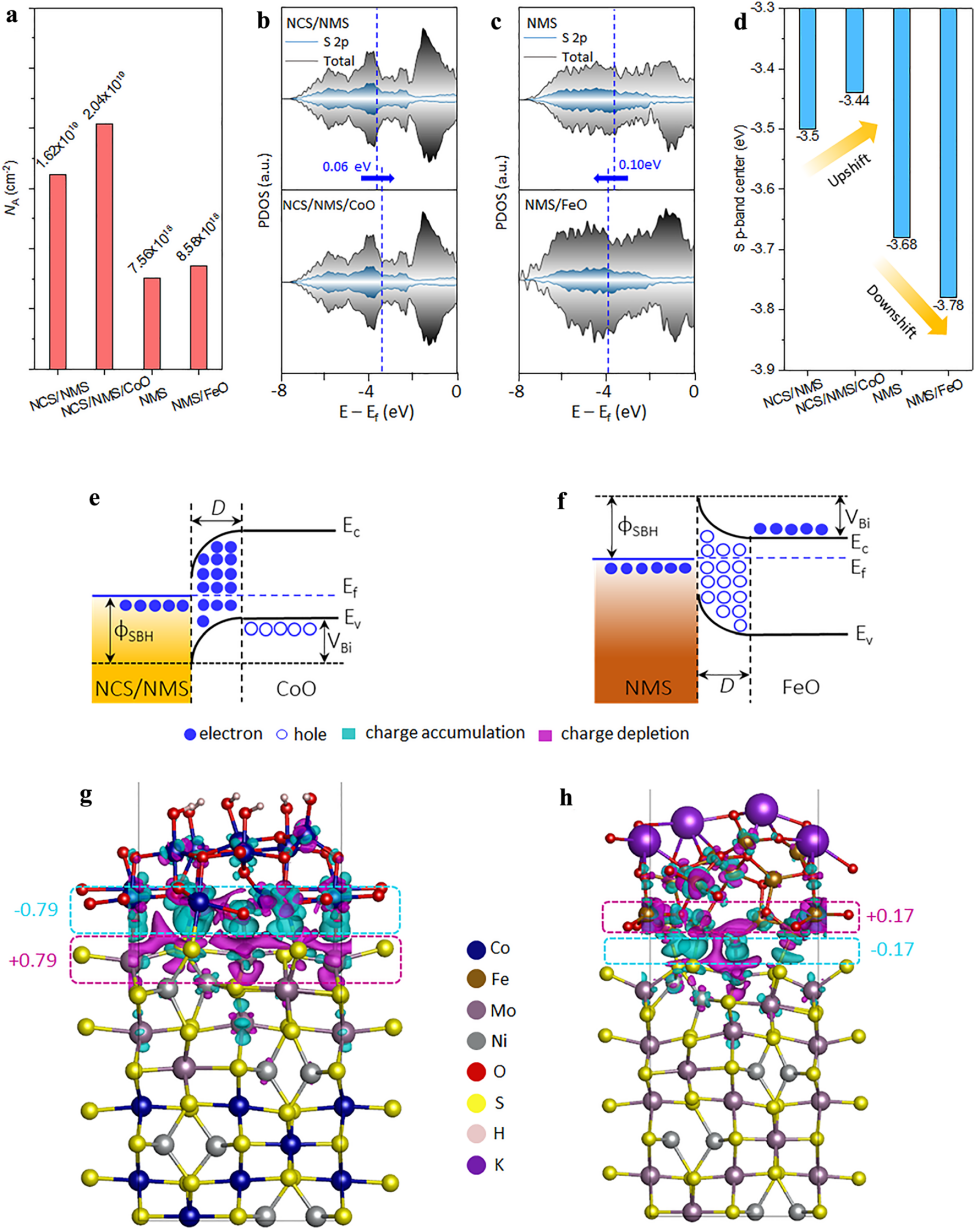
**a** Calculated number of active sites (*N*_A_) before and after the incorporation of the ALD layer. **b, c** Partial density of states for the S 2*p* orbital with the NCS/NMS, NCS/NMS/CoO, NMS, and NMS/FeO models. **d** Comparison of the S *p*-band center. Band alignment of a **e** bipolar NCS/NMS||CoO, and **f** unipolar NMS||FeO Schottky junction under equilibrium Charge density difference map for a **g** NCS/NMS||CoO, and **h** NMS||FeO Schottky junction at equilibrium

Both the intercalation and the surface redox pseudocapacitance mechanisms depend on the number of surface-active sites (*N*_A_) at the electrode–electrolyte interface. *N*_A_ was calculated using the reduction peak of each electrode (Fig. S15). Incorporation of the CoO and FeO layers significantly improved *N*_A_ (Fig. 6a). The optimized CoO and FeO layers were very thin and the aqueous electrolyte was able to permeate the transition metal oxides layers, so redox reactions occurred at the NCS/NMS-CoO and NMS-FeO interfaces and more deeply within the material. Therefore, NCS/NMS/CoO exhibited M–O (redox form CoOOH) as the first row of active sites and M‒S (redox form M‒SOH) as the second row. While NMS/FeO also had two rows of active sites. Increasing the thickness of the ALD layer can increase the number of M–O active sites in the first row, but this blocks the diffusion pathways to the M‒S active sites in the second row. Therefore, the optimization of the ALD layer thickness is vital in achieving the maximum *N*_A_. The thickness of the ALD layer can be precisely controlled by the number of ALD cycles [19]. The NCS/NMS and NMS electrodes were thus coated with CoO and FeO layers of different thicknesses, respectively, to optimize the interface kinetics (Table S4). The specific capacities of the NCS/NMS/CoO electrode with 100, 200, and 300 were then measured (Fig. S16a). At a low current rate, the specific capacity was independent of the number of ALD cycles but gradually became dependent as the current rate increased. The lower rate capability associated with 100 and 300 cycles indicates the presence of a low number of Co–O active sites in the first row and long/masked ion diffusion paths to the M‒S active sites in the second row, respectively. Similar results were obtained for different ALD cycles with the NMS negative electrode (Fig. S16b). Therefore, the participation of M‒S active sites is key to maintaining pseudocapacitive behavior at a high current rate, and the solid–solid interface (i.e., NCS/NMS-CoO and NMS-FeO) plays a crucial role in enhancing this behavior.

Theoretical calculations for the solid–solid junctions of the positive and negative electrodes were made to define the origin of the high-rate capability. The more stable and exposed surfaces of NCS(110), NMS(110), Co_3_O_4_(110) and Fe_2_O_3_(0001) were used for these calculations (Figs. S17 and S18). More details on surface and junction optimization are presented in Figs. S19 and S20. The calculated density of states (DOS) for NCS/NMS, NCS/NMS/CoO, NMS, and NMS/FeO is shown in Figs. S21 and S22. There was a greater number of electrons in the Fermi level (*E*_f_) which reflected the metallic nature of the NMS and NCS/NMS models. Furthermore, the CoO and FeO layers increased the DOS of the *E*_f_, indicating more favorable electronic properties, e.g., conductivity and electron mobility. Metallic NCS/NMS and CoO form a classical Schottky junction on contact because Co_3_O_4_ is a well-known p-type semiconductor. This is a bipolar Schottky junction because the major charge carriers on both sides are opposites (i.e., electrons and holes). Electronic equilibrium is achieved by transferring electrons from the NCS/NMS to the valance band of CoO; consequently, a hole depletion layer (i.e., an electron accumulation layer) is formed on the CoO side (Fig. 6e) [56]. Similarly, metallic NMS and n-type FeO form a unipolar Schottky junction with electrons as the major charge carriers on both sides (Fig. 6f). At equilibrium, electrons will flow to the NMS from the FeO conduction band and form a hole accumulation layer (i.e., an electron depletion layer) on the FeO side [57]. To verify this, the S *p*-band center for NMS and NCS/NMS was calculated before and after the addition of FeO and CoO layers. For the NCS/NMS/CoO model, the S *p*-band center shifted to the right, indicating electron-donor-like behavior (Fig. 6b). For the NMS/FeO model, S p-band center shifted to the left, indicating electron- acceptor-like behavior (Fig. 6c). Therefore, the electrical field created in the Schottky junction acts in the opposite direction for the positive and negative electrodes, as illustrated by the up and down shift of the S *p*-band center shown in Fig. 6d. This electric field can act as a driving force for the ion diffusion of oppositely charged ions (K^+^/OH^–^) during the charging and discharging processes [58].

Bader charge analysis was conducted to quantify the charge distribution in the Schottky junction. Two heterojunctions were modeled with K^+^ and OH^–^ ions at the edges to visualize the charge distribution under working conditions for NCS/NMS/CoO/OH^–^ (Fig. 6g) and NMS/FeO/K^+^ (Fig. 6h). The electron accumulation and depletion areas are shaded in cyan and magenta, respectively, with CoO losing 0.79 e and FeO gaining 0.17 e due to the formation of the Schottky junction. The charge distribution at the Schottky junction was more significant than at the electrode–electrolyte interface, meaning that the Schottky junction was responsible for the net current flow across the electrode–electrolyte interface. Schottky barrier height (ɸ_SBH_) and the voltage barrier (*V*_Bi_) are the two important parameters that control the flow of electrons through the Schottky junction. ɸ_SBH_ is a constant energy barrier that limits the further flow of electrons through the Schottky junction once equilibrium is reached. However, external alteration of *V*_Bi_ can cause electrons to flow in one direction depending on the type of Schottky junction [59].

**Fig. 7:**
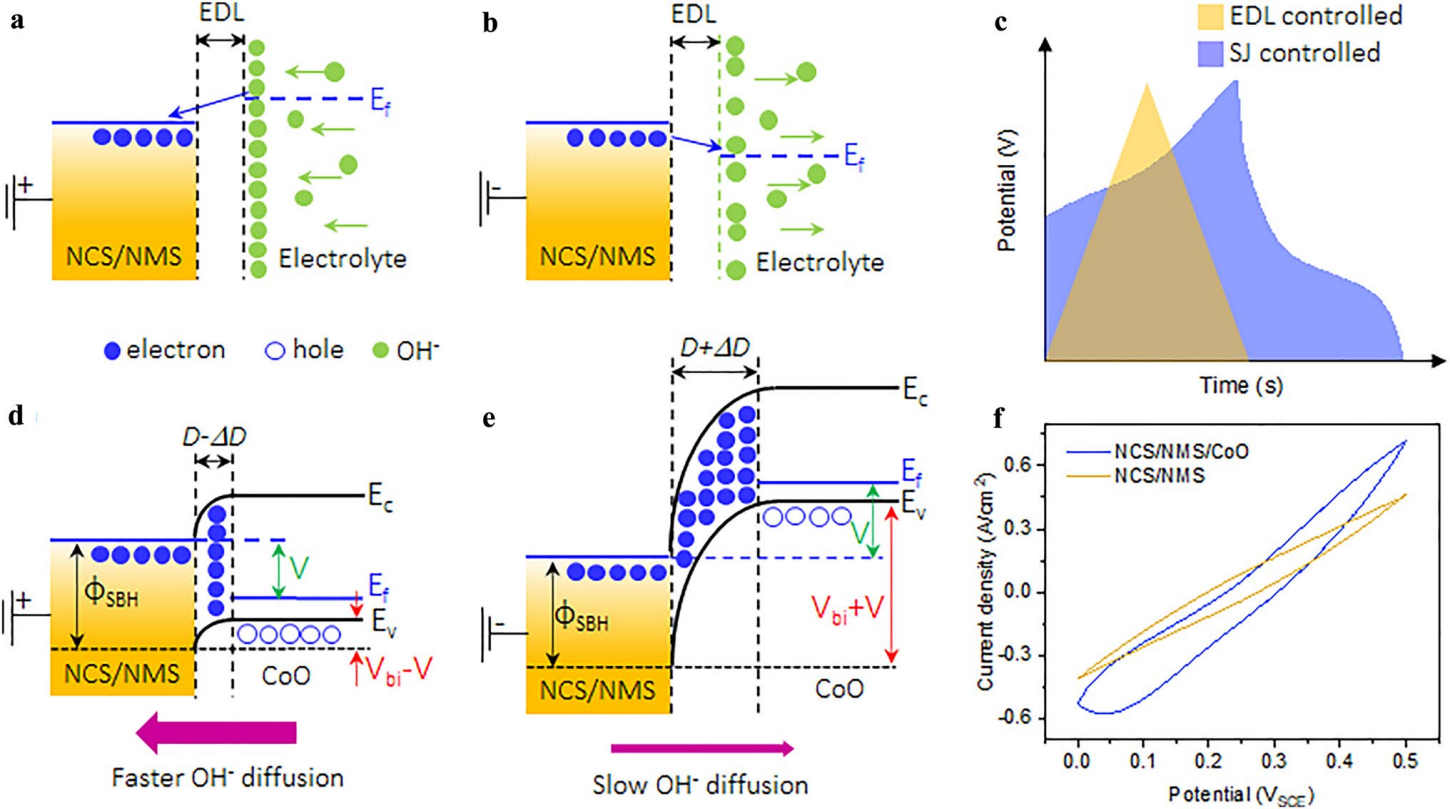
Band alignment for the NCS/NMS-electrolyte interface under **a** charging and **b** discharging **c** Schematic of the charge–discharge curves with ohmic and Schottky behavior. Band alignment for the NCS/NMS||CoO junction under **d** charging and **e** discharging **f** CV curves for bare and CoO deposited NCS/NMS positive electrodes at a scan ate of 100 mV s.^−1^

Under forward bias (i.e., charging), the NCS/NMS-electrolyte interface acts as a normal electrical double layer (EDL; Fig. 7a), where the current flow through the interface depends only on *R*_CT_ (which is inversely proportional to the applied bias) [60]. At a high current rate, there is no driving force for the diffusion of OH^–^ ions through the bulk, so most of the charge is stored via EDLC mechanism (Fig. 7c). Similarly, under reverse bias (i.e., discharging), OH- is rapidly repelled from the EDL by the electrons accumulating on the metal side (Fig. 7b). Thus, the likelihood of pseudocapacitive mechanism occurring is drastically reduced.

For the NCS/NMS/CoO electrode, V_Bi_ and the width of the depletion layer (*D*) become negligible while charging and the OH^–^ ions are attracted towards the junction by the holes on the CoO side (Fig. 7d). Therefore, more OH^–^ ions reach the M‒S active sites. While discharging, V_Bi_ and *D* become larger, limiting the release of OH^–^ from the bulk NCS/NMS side (Fig. 7e). Due to the slow release of OH^–^ ions from Schottky junction, the discharge time will be longer than with bare NCS/NMS. Unlike a normal EDL, the Schottky junction attracts and traps OH^–^ ions during the charging and discharging processes, respectively. In theory, this process is not affected by the current rate, but Schottky junction increases the likelihood of OH^–^ diffusion to the M‒S active sites at all current rates, which helps to maintain the pseudocapacitive behavior at high current rates. The CV curves for the NCS/NMS and NCS/NMS/CoO electrodes at a scan rate of 100 mV s^−1^ are compared in Fig. 7f. The NCS/NMS/CoO electrode demonstrated negligible deformation compared to the NCS/NMS electrode. The redox peaks visible after the addition of the CoO layer indicate that the Schottky junction improves the pseudocapacitive behavior at a high current rate. The impact of the unipolar Schottky junction on the NMS/FeO negative electrode is like that of the NCS/NMS/CoO positive electrode. The band alignment for NMS/FeO with respect to the charge and discharge conditions is displayed in Fig. S23. The opposite direction of the electric field formed in NMS||FeO junction attracts and traps K^+^ ions during the charging and discharging processes, respectively.

### Balanced Pseudo-capacitance of NCS/NMS/CoO||NMS/FeO Supercapattery Device

**Fig. 8:**
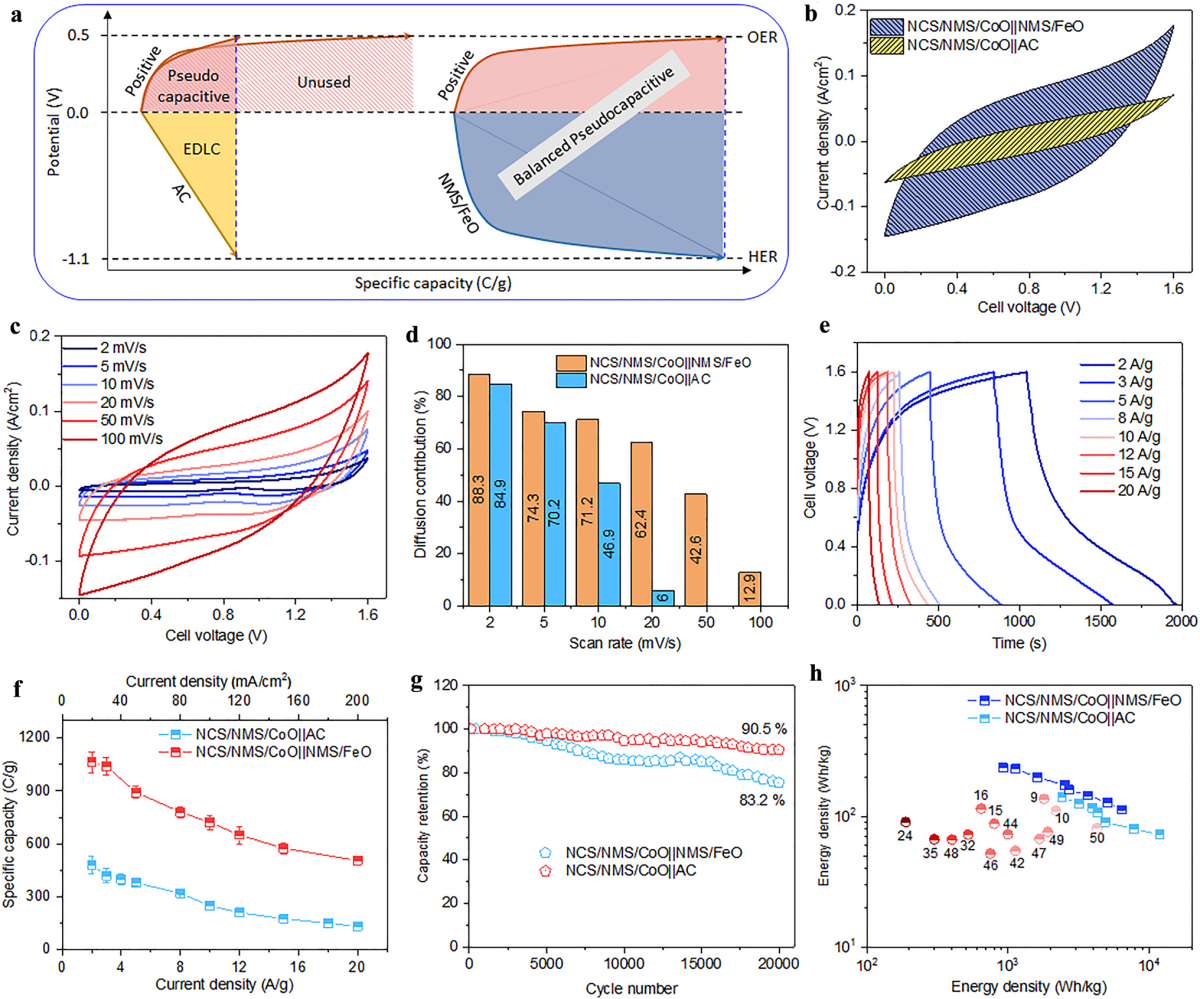
**a** Schematic of super capacity variation of the all solid-sate device depends on EDLC and pseudocapacitive negative electrode. **b** Comparison of CV curves of NCS/NMS/CoO||NMS/FeO and NCS/NMS/CoO||AC devices at 100 mV s^−1^. **c** CV curves of the NCS/NMS/CoO||NMS/FeO device at various scan rates. **d** Calculated contribution of the diffusion-controlled charge storage mechanism. **e** GCD curves of the NCS/NMS/CoO||NMS/FeO device. **f** Comparison of specific capacity. **g** Capacity retention, **h** Ragone plot with the comparison of recent reports tabulated in Table S4

As shown in Fig. 8a, the maximum specific capacity of a practical device can be limited by an unbalanced contribution of the positive and negative electrodes. Incorporating a positive pseudocapacitive and a negative EDLC electrode or vice versa in a practical device can compromise its overall specific capacity. Therefore, both the positive and negative electrode must have the same charge storage mechanism and a similar specific capacity range to ensure a balanced contribution [14]. Supercapattery devices were fabricated using pseudocapacitive NCS/NMS/CoO as a positive electrode and pseudocapacitive NMS/FeO or activated carbon (AC) as the negative electrode. AC is known for its superior EDLC behavior. The active mass of AC negative electrode was adjusted to be equal to that of the NMS/FeO negative electrode. The 2 M KOH-PVA gel electrolyte was used with stainless sheets as current collectors at both ends. PVA was selected as the polymer due to its excellent chemical stability, water solubility, non-toxicity, and biodegradability. PVA is a linear polymer that contains multiple OH- groups that absorb a large number of water molecules, thus improving the ionic conductivity of a solid electrolyte [61]. The comparison of CV curves of NCS/NMS/CoO||NMS/FeO and NCS/NMS/CoO||AC devices at 100 mV s^−1^ is shown in Fig. 8b. The smaller EDLC contribution of the AC negative electrode limited the overall charge storage of the NCS/NMS/CoO||AC device. The CV curves of the NCS/NMS/CoO||NMS/FeO supercapattery device obtained for the scan rate range of 2–100 mV s^−1^ is shown in Fig. 8c. It was evident that the full operating window of the NCS/NMS/CoO and NMS/FeO electrodes could be used for the solid-state device (1.6 V). The quasi-rectangular shape of the CV curves even at 100 mV s^−1^ scan rate indicates the sustained pseudocapacitive charge storage mechanism at high current rate operation. The calculated diffusion contribution (*C*_D_) to the total specific capacity (*C*_T_) of the supercapattery devices with NMS/FeO and AC negative electrodes is plotted against the scan rate in Fig. 8d. The NMS/FeO negative electrode significantly increased the diffusion process at all scan rates compared to the bare AC negative electrode and maintained the battery behavior at ~ 12.86% even at 100 mV s^−1^.

Figure 8e shows the GCD curves of the NCS/NMS/CoO||NMS/FeO device at current densities in the range of 3–20 A g^−1^. The nonlinear profile of the charging and discharging curves denotes the pseudocapacitive charge storage mechanism. The *IR* drop in the GCD curve was negligible, suggesting excellent material conductivity and low interface resistance in the fabricated device. The NCS/NMS/CoO||NMS/FeO supercapattery device achieved the highest specific capacity value of 1062.62 C g^−1^ at 2 A g^−1^ as compared to other devices described in the literature. In contrast, the NCS/NMS/CoO||AC device achieved a specific capacity of only 480.12 C g^−1^ at 2 A g^−1^ (Fig. 8f). Such a high throughput electrochemical performance can be attributed to the balanced contribution of positive and negative electrodes to support the pseudocapacitive charge storage mechanism.

The cycling stabilities of both supercapattery devices were estimated by GCD curves obtained at 10 A/g current density for 20,000 cycles. As shown in Fig. 8g, the NCS/NMS/CoO||AC and NCS/NMS/CoO||NMS/FeO devices retained 83.2% and ~ 90.5% of their original specific capacities, respectively. Figure 8h represents a Ragone plot of gravimetric energy and power density calculated using the total mass of the active material. The NCS/NMS/CoO||NMS/FeO and NCS/NMS/CoO||AC devices obtained energy densities of 236.14 and 140.82 Wh kg^−1^ with power densities of 921.94 and 2417.94 W kg^−1^, respectively. These values fall in the supercapattery zone of the Ragone plot and exceed those of all supercapacitors reported in the literature. For comparison, see Table S5. The EIS analysis results of the fabricated supercapattery devices are shown in Fig. S24, which was fitted with the equivalent circuit in Fig. S11b. The fitted results are shown in Table S6. The observed R_CT_ for NCS/NMS/CoO||NMS/FeO and NCS/NMS/CoO||AC devices was 7.31 and 17.54 Ω, respectively. The higher *C*_dl_ value without any Warburg component (W) at the low frequency region of the NCS/NMS/CoO||AC device shows the dominance of EDLC behavior and poor diffusion of electrolyte ions in bulk AC [62]. The Warburg component of the NCS/NMS/CoO||NMS/FeO device suggests strong electrolyte ion diffusion in the electrode bulk structure. The self-discharge of NCS/NMS/CoO||NMS/FeO device is shown in Fig. S25. The device retained 43.75% of its initial cell voltage up to 48 h at 25 °C. Two NCS/NMS/CoO||NMS/FeO devices were connected in series with the total output voltage of 3.2 V to demonstrate their practical application prospects. Initially, the two devices connected in series were charged for 20 s and connected to different colored light emitting diodes such as red (1.6 V), yellow (1.8 V), blue (2.5–3.0 V), and white (3.0 V) during discharge (Fig. S26). The intensity of light emitted at the initial stage and the slow long-term fading indicated the high energy density of the fabricated device. Additionally, a small motor (3 V, 750 mA) was operated by the charged device, showing the excellent energy output of the NCS/NMS/CoO||NMS/FeO device.

## Conclusion

In summary, we show that the poor pseudocapacitive behavior of supercapacitor electrodes can be strategically alleviated by incorporating a Schottky junction next to the electrode–electrolyte interface through atomic layer deposition. The Schottky junction attracts and traps the intercalation ions (OH^–^ or K^+^) during the charging and discharging processes, respectively, to improve the pseudocapacitive behavior at higher current rate. With improved surface wettability and Schottky junction-controlled ion diffusion, positive and negative electrodes recorded new benchmark specific capacities. By balancing the specific capacities of both electrodes, an energy density of 236.14 Wh kg^−1^ was achieved with a total active mass of 15 mg cm^−2^ in a solid-state device with 90.5% capacity retention after 20,000 charge–discharge cycles. This NCS/NMS/CoO||NMS/FeO device developed using proposed strategy illustrates the potential for the development of supercapacitors that adapt well to the supercapattery zone of a Ragone plot. This strategy is applicable various electrochemical energy storage and conversion processes and is suitable for large-scale operation.

## Supporting Information

Additional XRD data, XPS analysis, *J–V* curves, HR-SEM images, and electrochemical analysis results.

### Supplementary Information

Below is the link to the electronic supplementary material.

Supplementary file1 (PDF 2408 KB)
