# Supplementary material for: Boosting Pseudocapacitive Behavior of Supercapattery Electrodes by Incorporating a Schottky Junction for Ultrahigh Energy Density
Source: Nanomicro Lett. 2023 Mar 10;15:62. doi: 10.1007/s40820-023-01016-6 (PMC10006391; doi:10.1007/s40820-023-01016-6)
Supplement: Supplementary file 1 — Supplementary file1 (PDF 2408 KB) [file 40820_2023_1016_MOESM1_ESM.pdf]

Supporting Information for

Boosting Pseudocapacitive Behavior of Supercapattery Electrodes by Incorporating a Schottky Junction for Ultrahigh Energy Density

Selvaraj Seenivasan[1], Kyu In Shim[2], Chaesung Lim[3], Thangavel Kavinkumar[1], Amarnath T. Sivagurunathan[1], Jeong Woo Han[2, 3, *], and Do-Heyoung Kim[1, *]

[1]School of Chemical Engineering, Chonnam National University, 77 Yongbong-ro, Gwangju 61186, Republic of Korea

[2]Division of Environmental Science and Engineering, Pohang University of Science and Technology (POSTECH), Pohang, 37673, Republic of Korea

[3]Department of Chemical Engineering, Pohang University of Science and Technology (POSTECH), Pohang, 37673, Republic of Korea

*Corresponding authors. E-mail: jwhan@postech.ac.kr (Jeong Woo Han); kdhh@chonnam.ac.kr (Do-Heyoung Kim)

Supplementary Figures and Tables

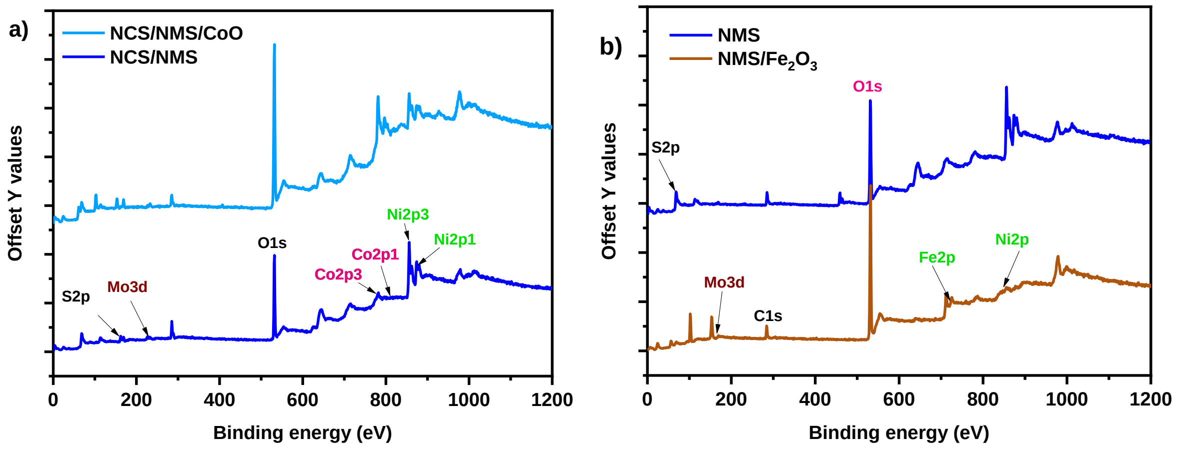

Fig. S1 (**a**) Survey spectra of $NiCo_2S_4/NiMo_2S_4$ (NCS/NMS) and $NiCo_2S_4/NiMo_2S_4/Co_3O_4$ (NCS/NMS/CoO). (**b**) Survey spectra of $NiMo_2S_4$ (NMS) and $NiMo_2S_4/Fe_2O_3$ (NMS/FeO)

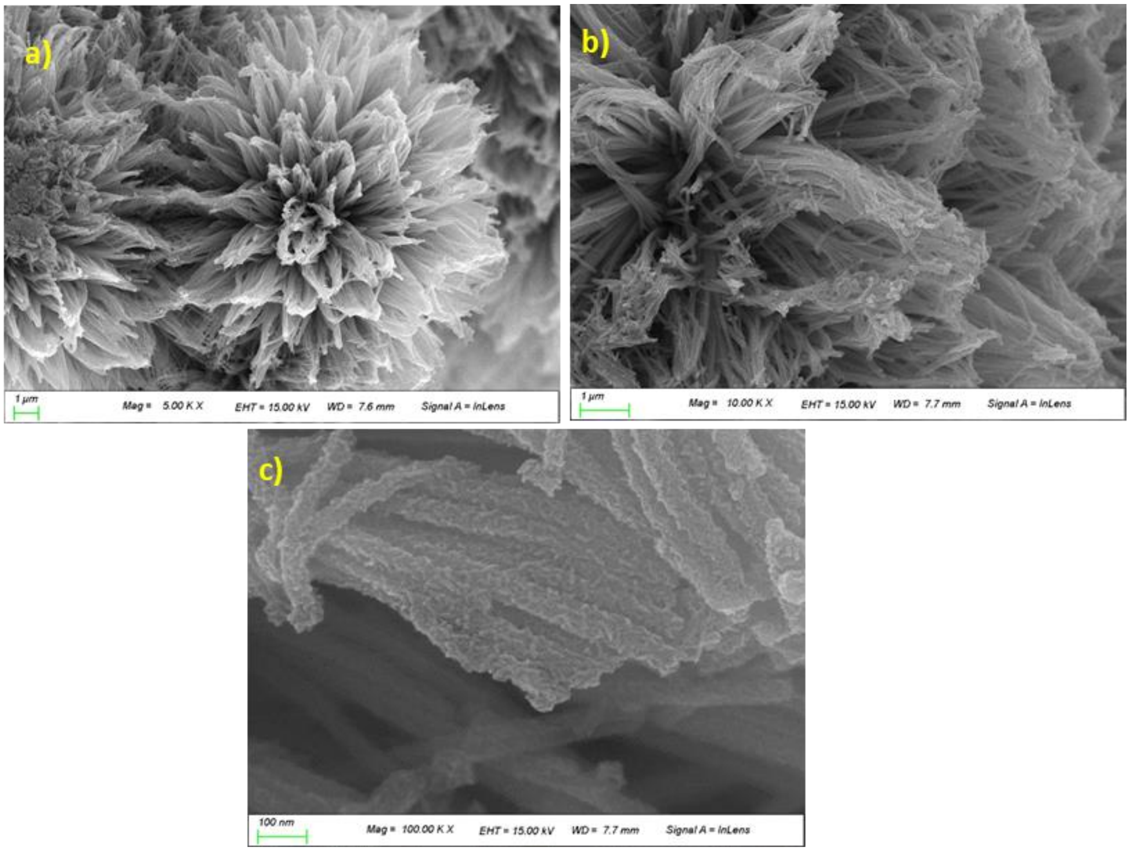

Fig. S2 (**a-c**) High-resolution SEM images of NCS grown on Ni foam

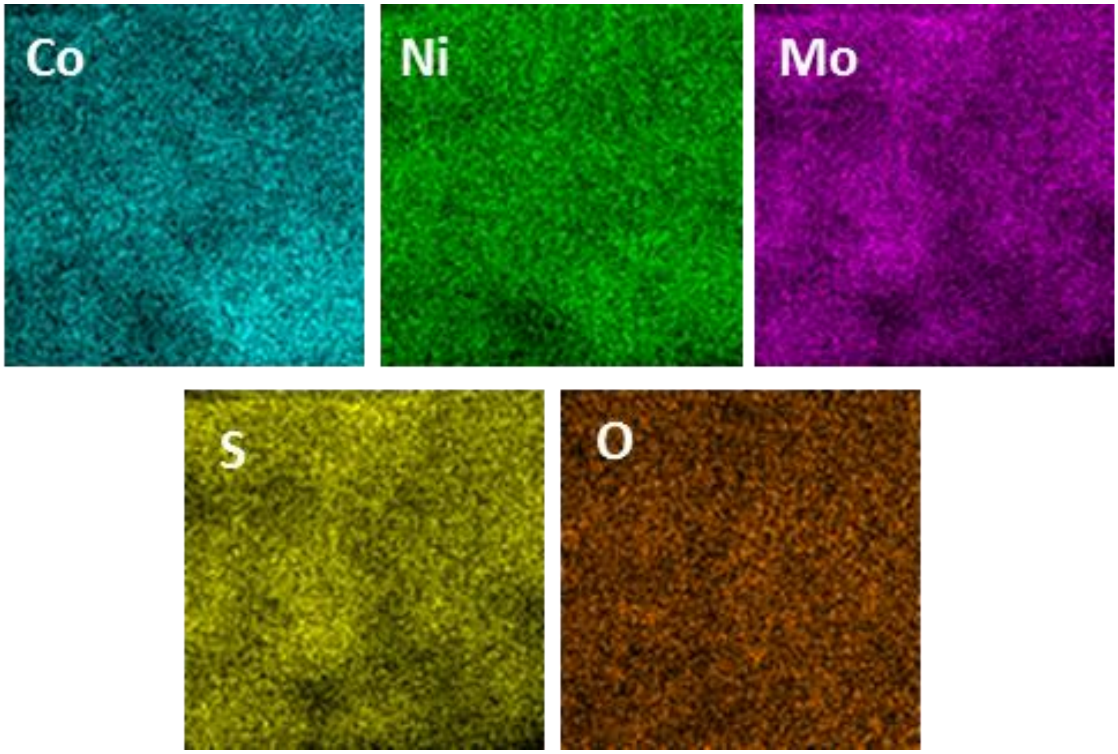

Fig. S3 Scanning transmission electron microscopy-energy dispersive spectroscopic (STEM-EDS) images of the NCS/NMS/CoO electrode

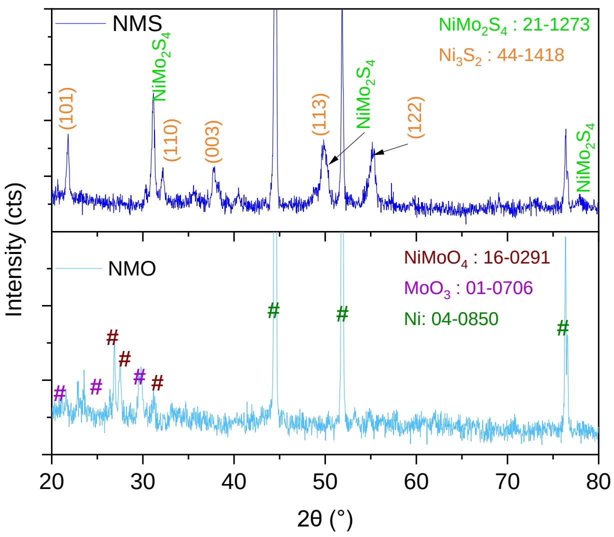

Fig. S4 XRD spectra of NMO and NMS electrodes

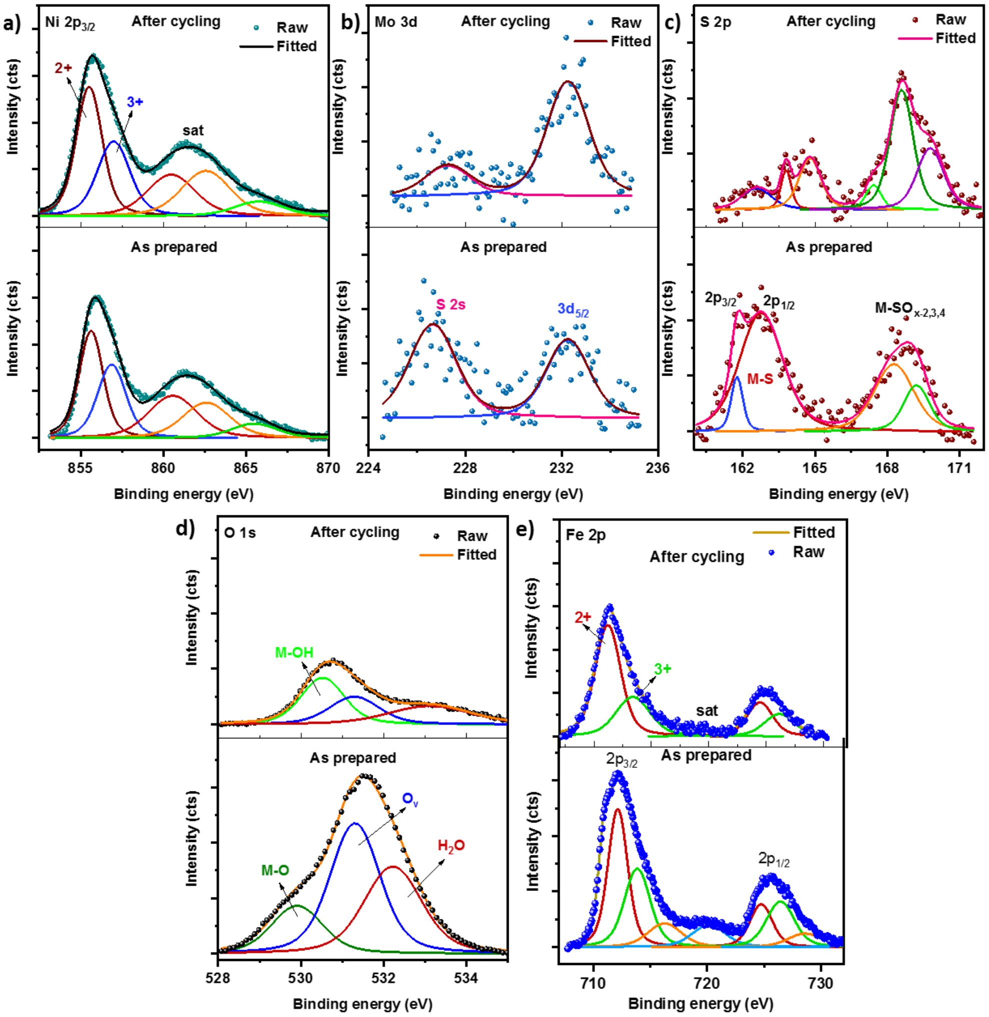

Fig. S5 XPS spectra of the NMS/FeO electrode before and after 20,000 GCD cycles at 10 A/g; (**a**) Ni 2p, (**b**) Mo 3d, (**c**) S 2p, (**d**) O 1s, and (**e**) Fe 2p

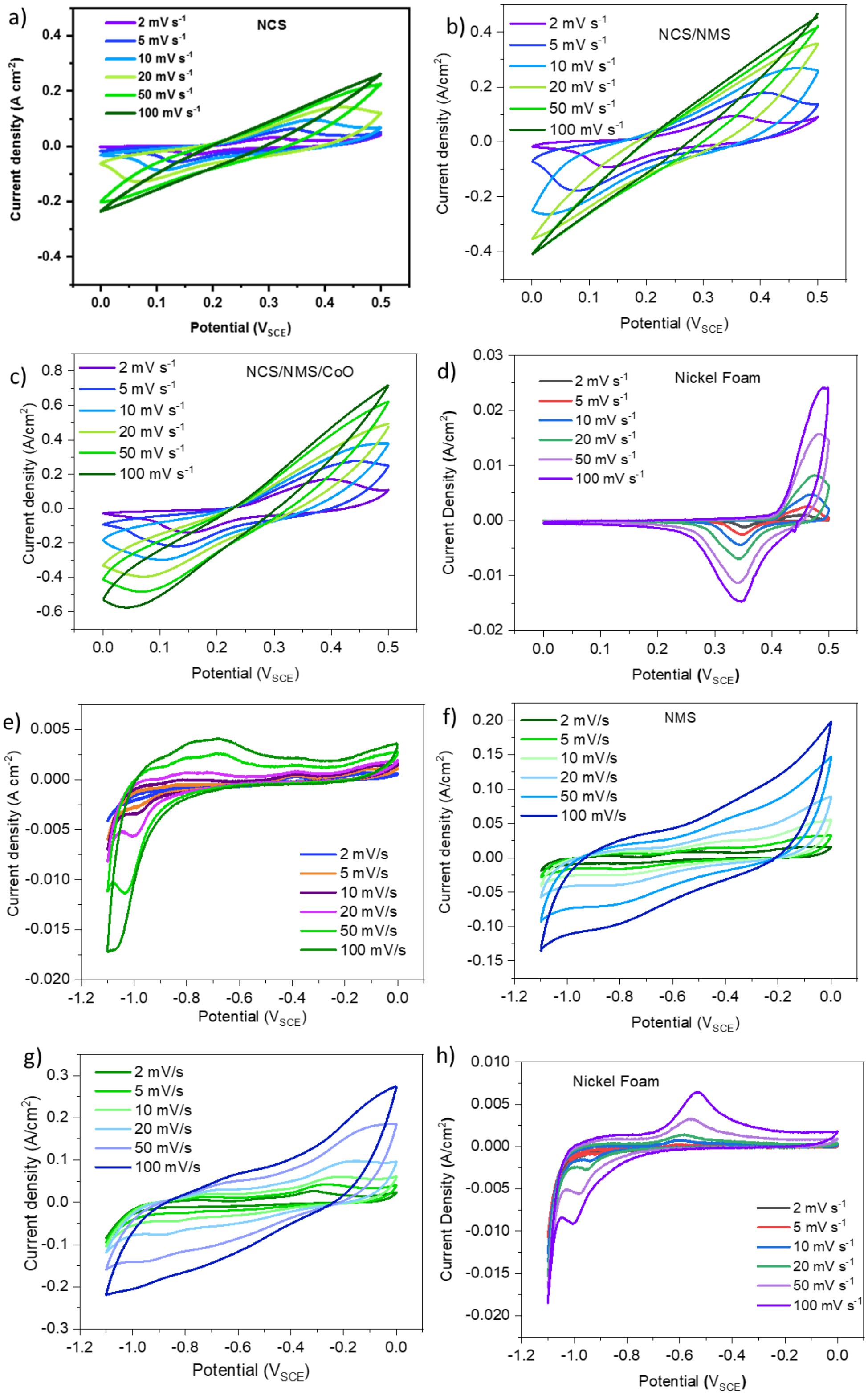

Fig. S6 CV plots of (**a**) NCS, (**b**) NCS/NMS, (**c**) NCS/NMS/CoO, (**d**) bare Ni foam under positive potentials. CV plots of (**e**) NMO, (**f**) NMS, (**g**) NMS/FeO, and (**h**) bare Ni foam under negative potentials at various scan rates

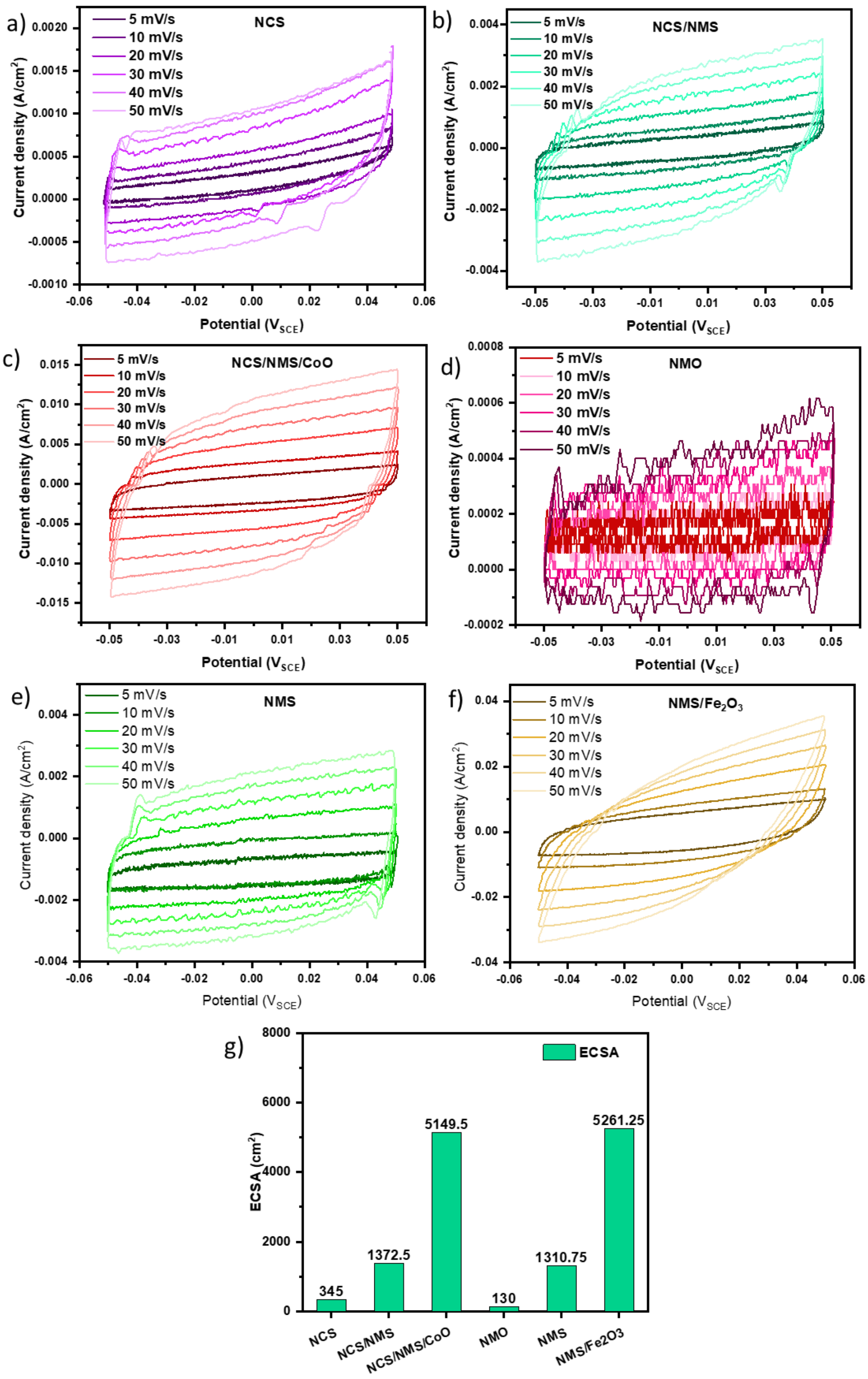

Fig. S7 (**a**–**f**) CV measurements of NCS, NCS/NMS, NCS/NMS/CoO, NMO, NMS, and NMS/FeO at scan rates of 5, 10, 20, 30, 40, and 50 mV/s in 2 M KOH electrolyte. (**g**) Comparison of measured ECSA values

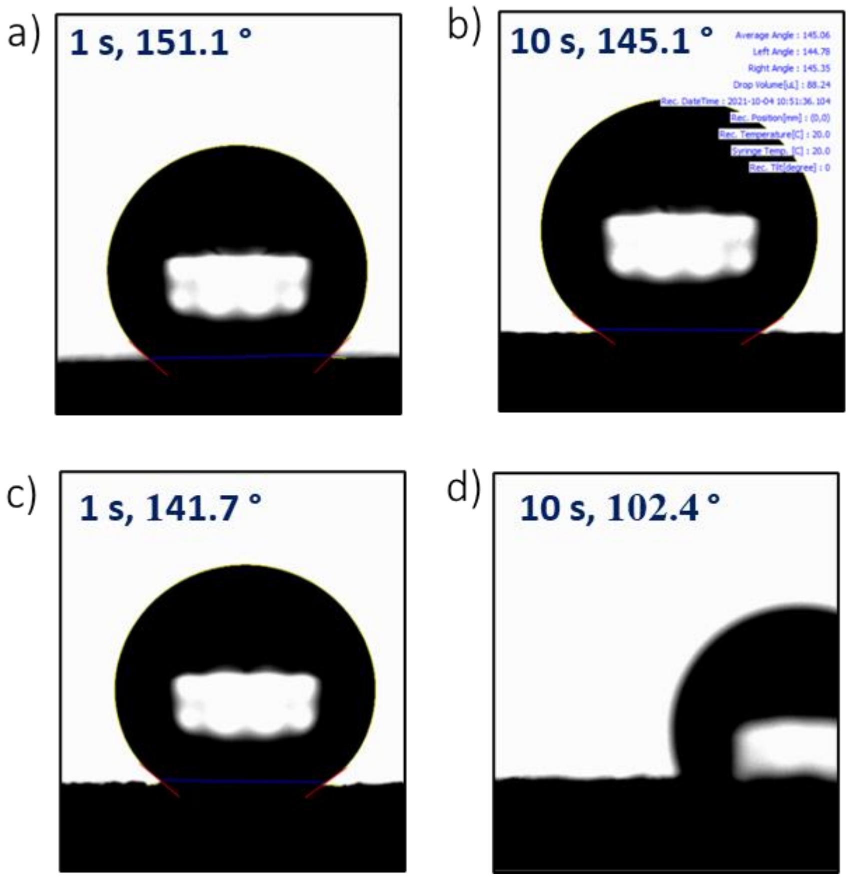

Fig. S8 Contact angle (θ) measurement of (**a, b**) NCS/NMS, and (**c, d**) NCS/NMS/CoO electrodes

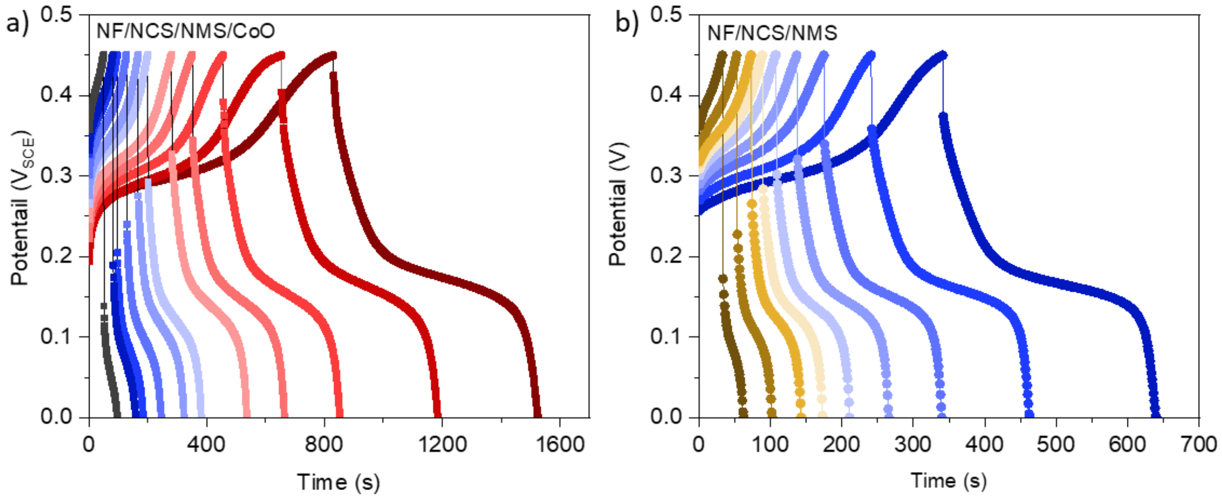

Fig. S9 GCD curves of (**a**) NCS/NMS/CoO, and (**b**) NCS/NMS positive electrodes at various current densities

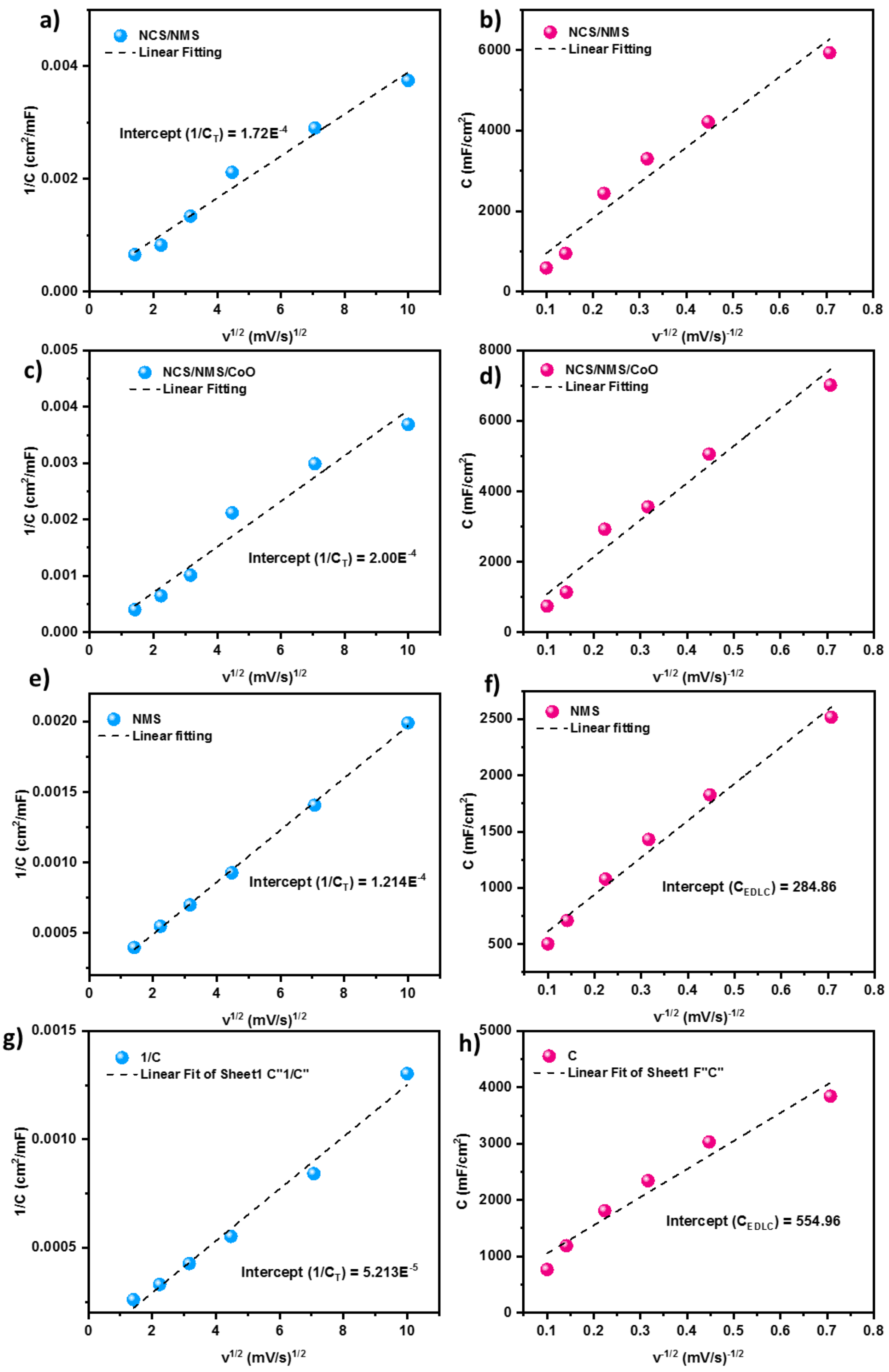

Fig. S10 (**a, c, e, g**) Plots of reciprocal of areal capacitance (1/*C*) versus square root of scan rates ($v^{1/2}$) and (**b, d, f, h**) plots of gravimetric capacitance (*C*) versus reciprocal of square root of scan rate ($v^{-1/2}$) for NCS/NMS, NCS/NMS/CoO, NMS, and NMS/FeO electrodes

Trasatti Method

The contributions of the pseudocapacitive and electrical double layer mechanisms are typically calculated using the Trasatti method. First, the areas of the CV curves at various scan rates are evaluated and the capacitance is calculated using the following formula:

$$C = \frac{A}{2\,\Delta V\,v} \tag{S1}$$

where C is the areal capacitance (mF/cm^2), ΔV refers to the potential window (V), and A is the area enclosed by the CV curves (mAV/cm^2) at different scan rates v (V/s). Assuming a semi-infinite diffusion pattern of ion diffusion, a linear relationship can be derived between the reciprocal of areal capacitance ($1/C$) and square root of scan rates ($v^{1/2}$) as follows:

$$\frac{1}{C} = \frac{1}{C_T} + cv^{1/2} \tag{S2}$$

where C_T is the maximum capacitance (mF/cm^2), the sum of the contributions of the pseudocapacitive and electrical double layer mechanisms, and c is a constant. The electrical double layer contribution can be calculated using the following relation,

$$C = C_{EDLC} + cv^{-1/2} \tag{S3}$$

The intercept obtained from linear fitting of the above relationship gives the value of the electrical double layer contribution at the maximum areal capacitance. The subtraction of C_{EDLC} from C_T yields the maximum pseudocapacitance (C_D).

$$C_D = C_T - C_{EDLC} \tag{S4}$$

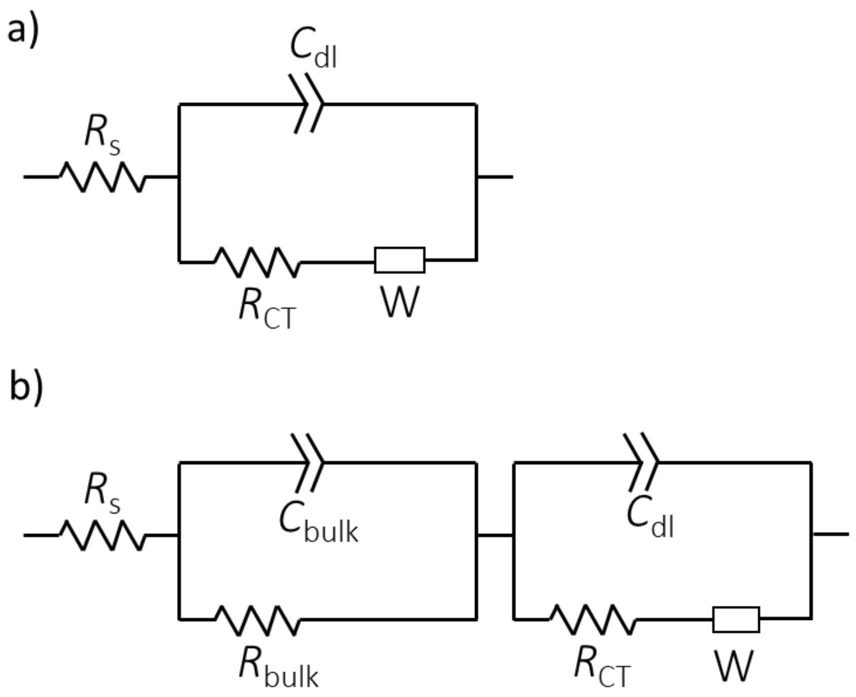

Fig. S11 (**a**) Equivalent circuit used to fit the data in (**a**) Fig. 4h and Fig. 5h, and (**b**) Fig. S24

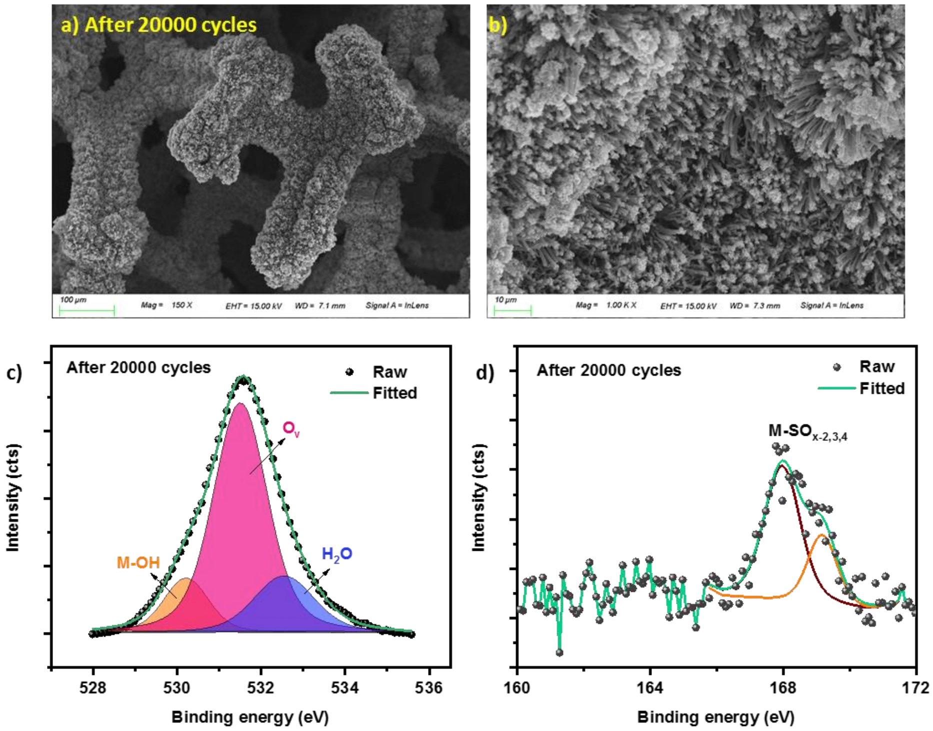

Fig. S12 (**a, b**) HR-SEM images of NCS/NMS/CoO electrode after 20,000 GCD cycles at 10.8 A/g current density, and high resolution XPS profiles of (**c**) O 1s and (**d**) S 2p

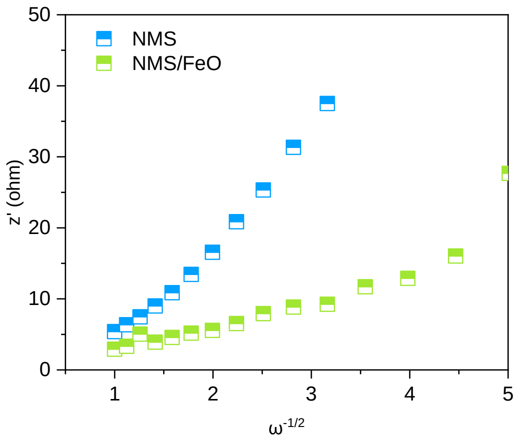

Fig. S13 Plot of real impedance and reciprocal of square root of angular frequency from EIS spectra of NMS and NMS/FeO

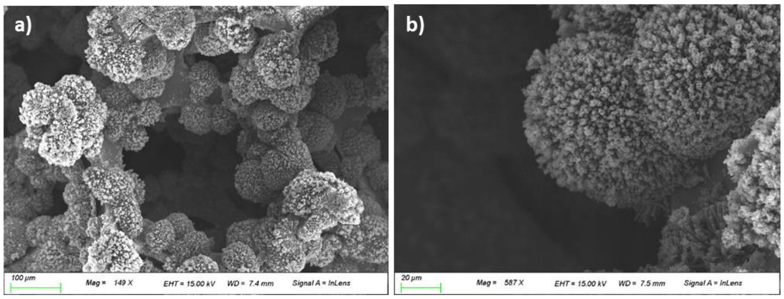

Fig. S14 HR-SEM images of the NMS/FeO negative electrode after 20,000 GCD cycles

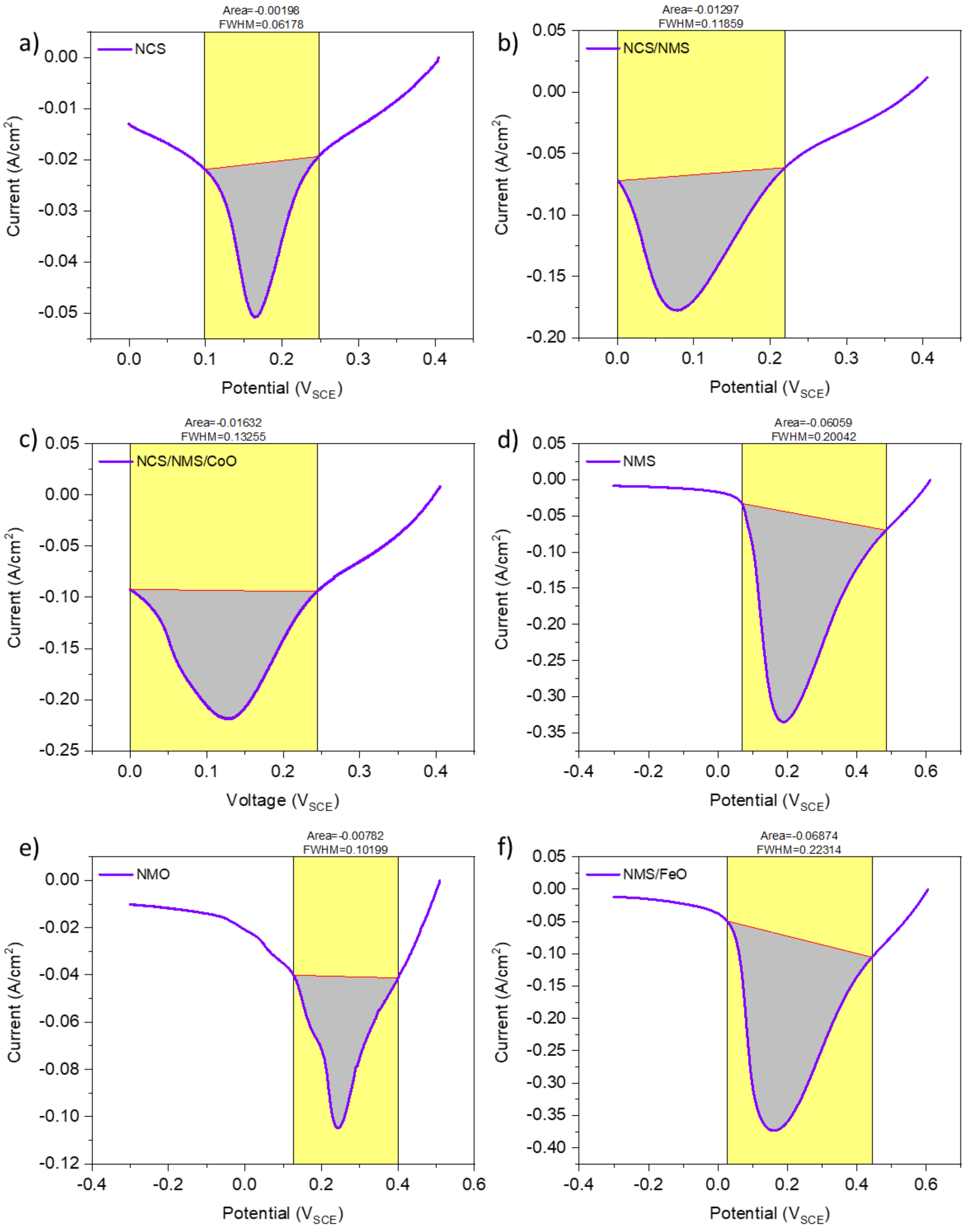

Fig. S15 Reduction peak of (**a**) NCS, (**b**) NCS/NMS, and (**c**) NCS/NMS/CoO positive electrodes at 5 mV/s. (**d**) NMS, (**e**) NMO, and (**f**) NMS/FeO negative electrodes at 50 mV/s

Calculation of Number Surface Active Sites (N_A)

Associated charge with the reduction peak (Q) can be calculated using the following expression: [S1]

$$Q = \frac{\int I\, dV}{v} \quad \text{(S5)}$$

where Q (C) is the total charge associated with the reduction peak and v (V/s) is the scan rate. For simplicity, we assume that all the surface redox reactions are single electron transfer reactions. Then, the number of electrons calculated above is the number of surface active sites (N). $q = 1.602 \times 10^{-19}$ C.

$$N = \frac{Q}{q} \quad \text{(S6)}$$

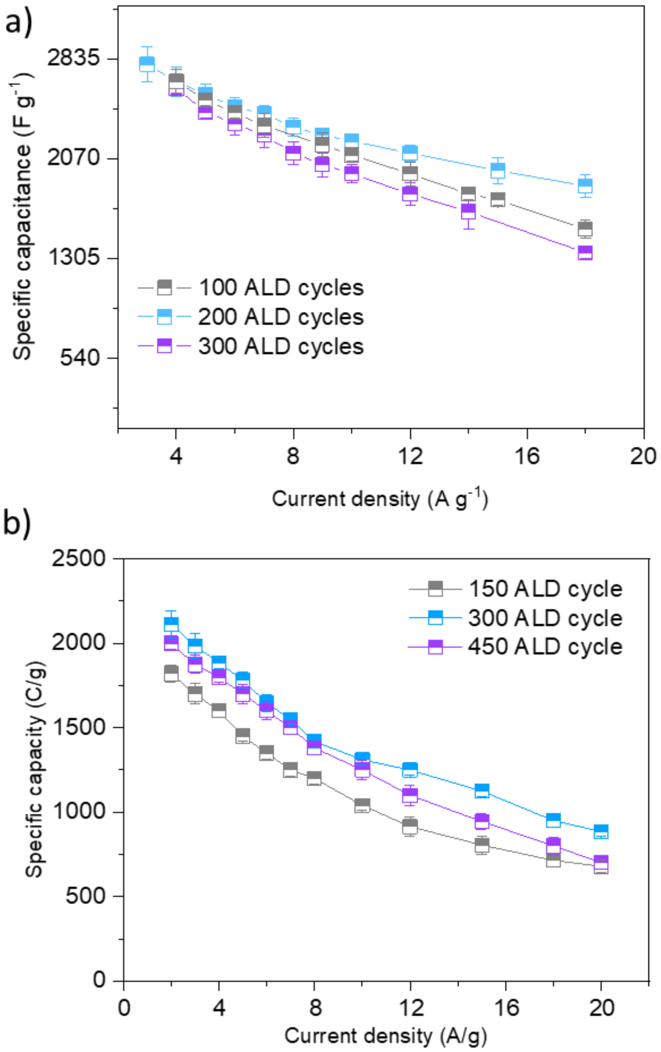

Fig. S16 (**a**) Specific capacity of the NCS/NMS/CoO electrode after 100, 200, and 300 ALD cycles. (**b**) Specific capacity of the NMS/FeO electrode after 150, 300, and 450 ALD cycles

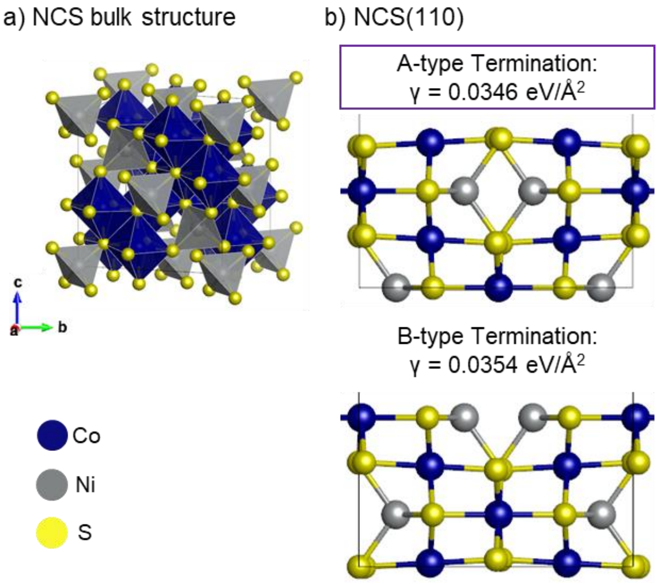

Fig. S17 Bulk structures and terminations of (**a, c**) NCS and (**b, d**) NMS samples

The DFT-optimized lattice parameters of spinel $NiCo_2S_4$ (NCS) were a = b = c = 9.33 Å. The more stable A-type termination was selected by comparing the surface energy (γ) of A-type and B-type termination. NMS(110) facet was adopted since hydrothermal synthesis was utilized.

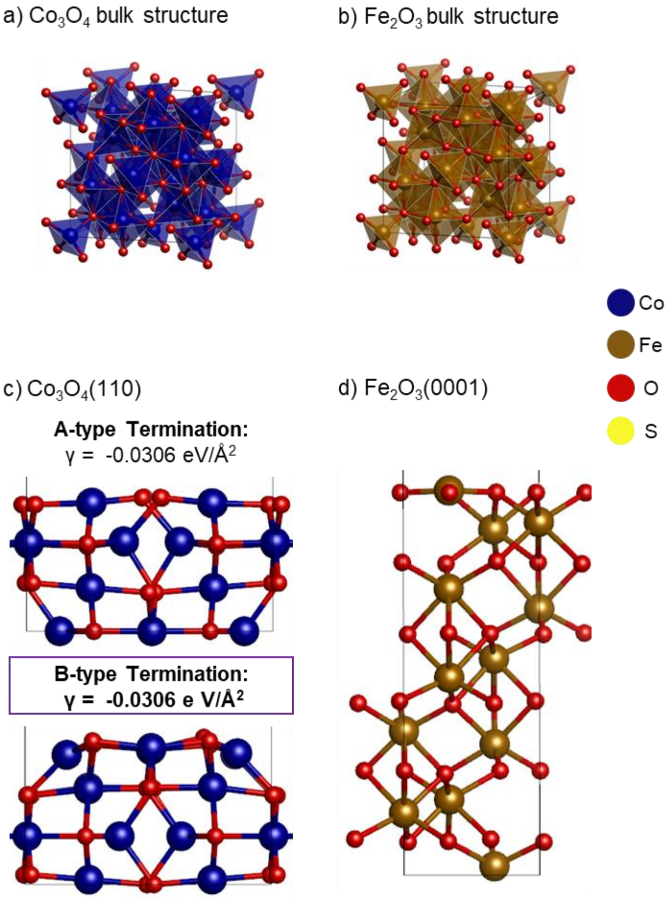

Fig. S18 Bulk structures and terminations of (**a, c**) CoO and (**b, d**) FeO overlayers

The DFT-optimized lattice parameters of spinel Co_3O_4 were $a = b = c = 8.15$ Å. According to the literature, Co_3O_4(110) facet was selected as an active surface. (110) facet is dominant and known to have a high density of exposed Co atoms [S2, S3]. The more stable B-type termination was selected by comparing the surface energy (γ) of A-type and B-type termination. The DFT-optimized lattice parameters of spinel α−Fe_2O_3 were a = b = 5.10 Å and c = 13.88 Å, and Fe−O_3−Fe-termination was selected since it is known as the most stable termination.

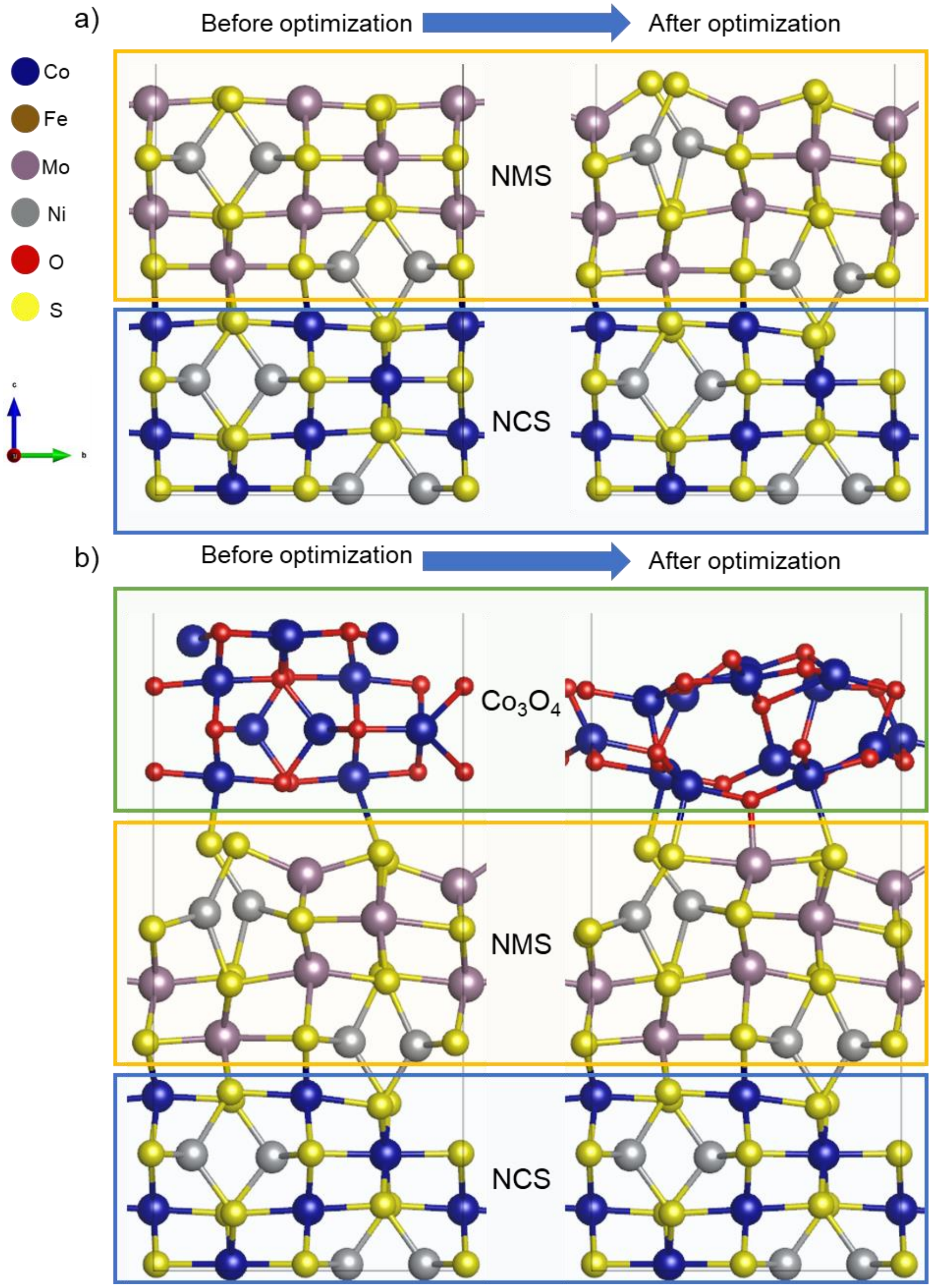

Fig. S19 (**a**) NCS/NMS(110) heterostructure was optimized. (**b**) Optimized junction of NCS/NMS/CoO hybrid model

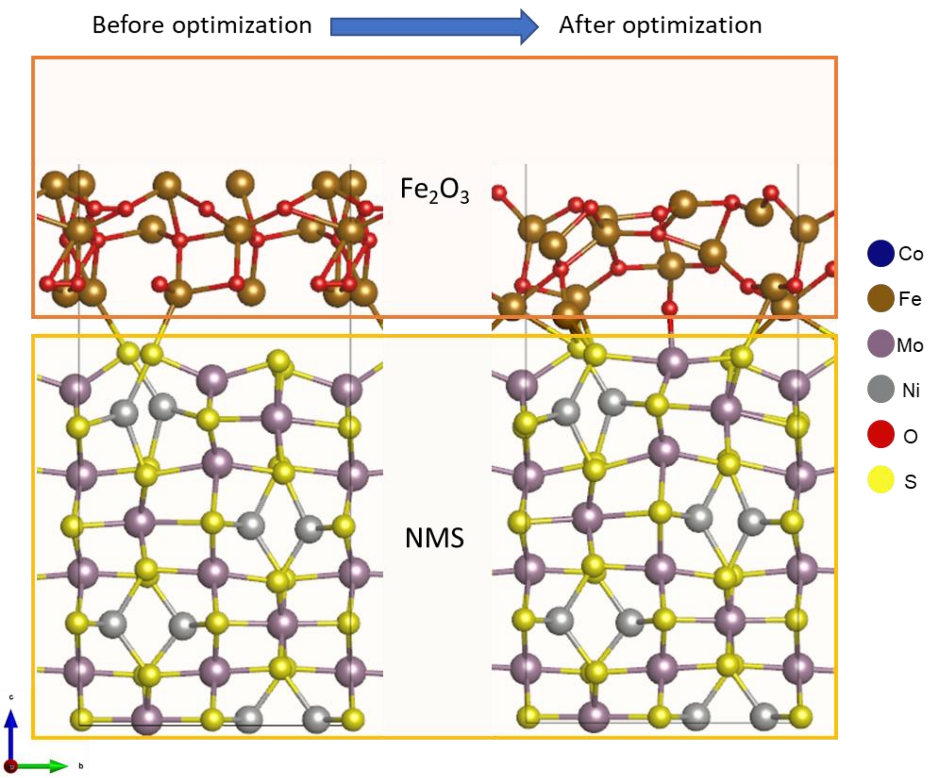

Fig. S20 Optimized junction of NMS/FeO hybrid model

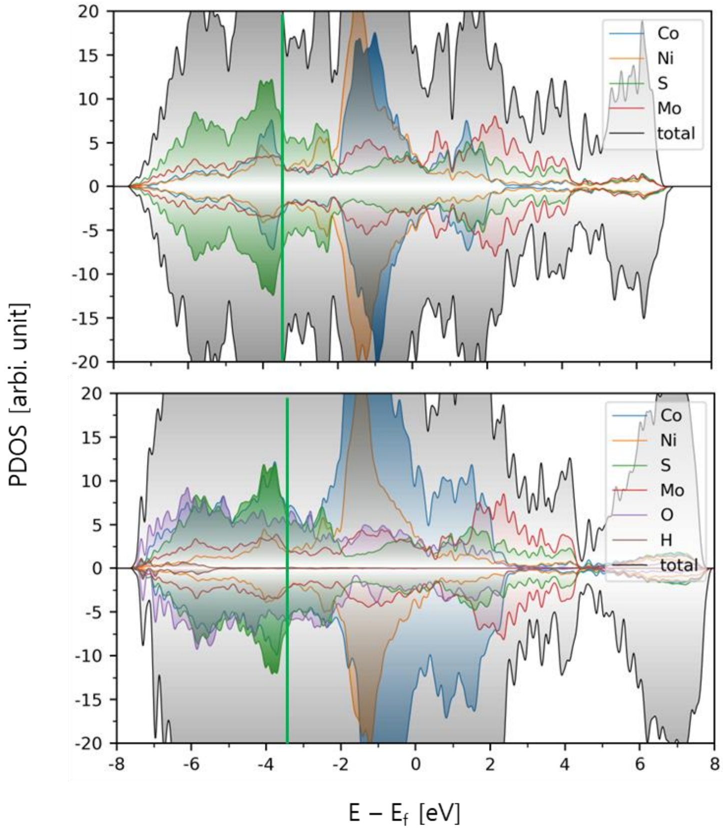

Fig. S21 Partial density of states of NCS/NMS and NCS/NMS/CoO/OH^- heterostructure models

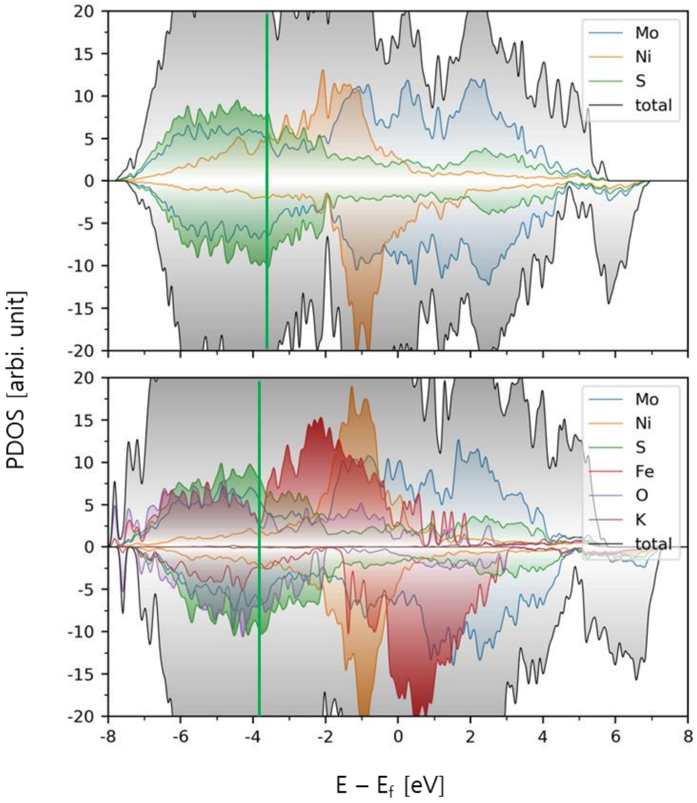

Fig. S22 Partial density of states of NMS and NMS/FeO/K^+ heterostructure models

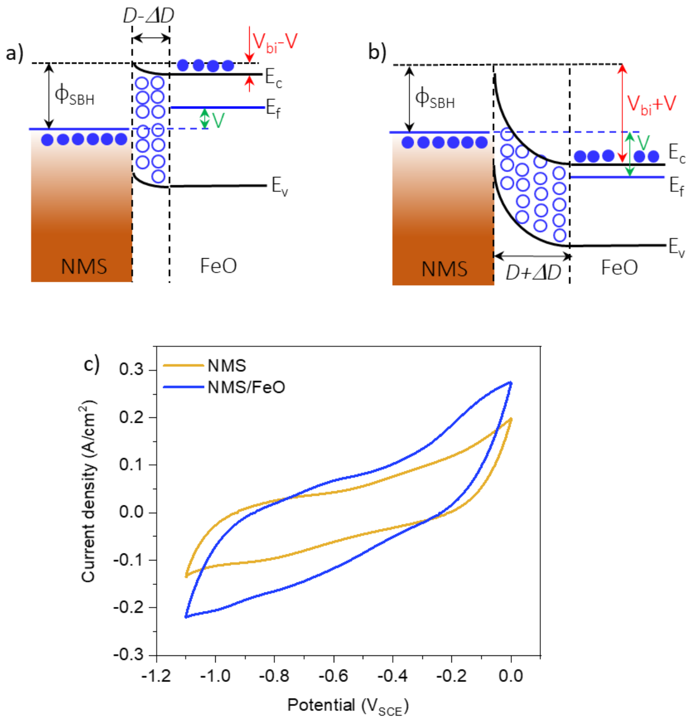

Fig. 23 Band alignment of NMS||FeO junction under (**a**) charging and (**b**) discharging conditions. (**c**) CV curves of bare and FeO deposited NMS negative electrode at 100 mV/s scan rate

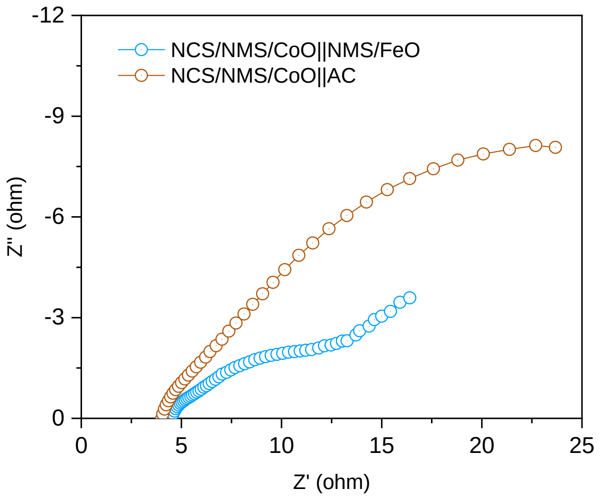

Fig. S24 Nyquist plot of all solid-state NCS/NMS/CoO||NMS/FeO and NCS/NMS/CoO||AC devices measured at 0.5 V versus open circuit voltage

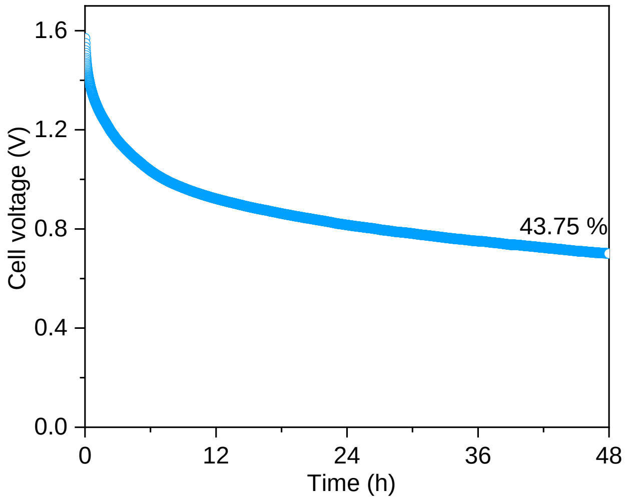

Fig. S25 Voltage retention of NCS/NMS/CoO||NMS/FeO supercapattery device

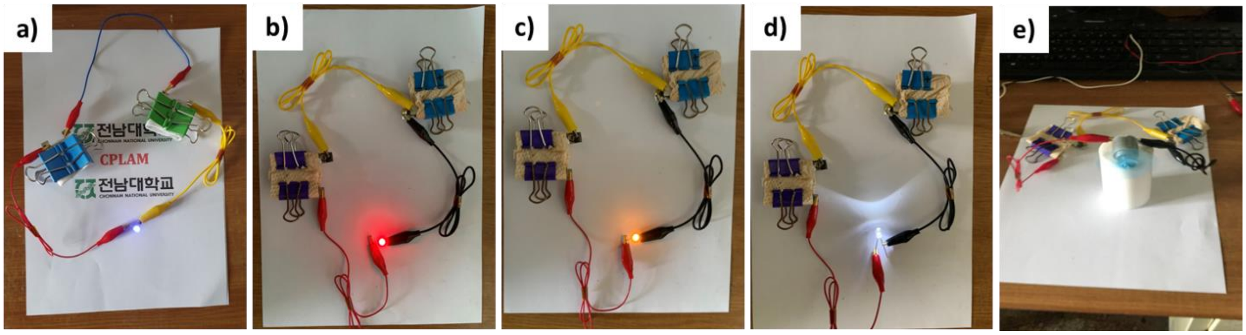

Fig. S26 Digital photograph of (**a**) blue, (**b**) red, (**c**) yellow, (**d**) white LED and (**e**) electric fan powered by two NCS/NMS/CoO||NMS/FeO supercapattery devices in series

Table S1 Comparison of the electrochemical performances of recently reported transition metal-based positive electrodes

No.	Material	Mass loading (mg/cm^2)	Electrolyte	Specific capacitance, F/g, or Capacity, C/g (Current density, A/g)	Stability (Cycles)	Refs.
1	$NiCo_2S_4/NiMo_2S_4/Co_3O_4$	10	2 M KOH	6,210 F/g or 2794.5 C/g (3.0 A/g)	94.3% (20,000)	This work
2	$NiCo_2S_4$	2	1 M KOH	2,206 F/g (4 A/g)	94.6% (2,000)	[S4]
3	CuS/rGO	0.75	6 M KOH	2,317.8 F/g (1 A/g)	96.2% (1,200)	[S5]
4	WS_2/rGO	10	1 M KOH	2,508.07 F/g (1 mV/s)	89.6% (5,000)	[S6]
5	MnS/rGO	0.9	3 M KOH	2,220.07 F/g (0.5 A/g)	92.1% (1,000)	[S7]
6	Bi_2S_3/MoS_2	-	6 M KOH	3,040 F/g (1 A/g)	92.6% (5,000)	[S8]
7	CoS_2-Co_9S_8/Mo_2S_3	0.5	3 M KOH	2,777.5 F/g (1 A/g)	100% (8,000)	[S9]
8	$NiCo_2O_4/MoO_2$/NiO	4.2	2 M KOH	1,732.5 C/g (2 A/g)	97% (20,000)	[S10]
9	rGO/$CuCo_2O_4/Ni_4Mo/MoO_2/Co_3O_4$	4.4	2 M KOH	2,334.8 F/g (1 A/g)	92.3% (20,000)	[S11]
10	$CuCo_2O_4/NiMoO_4$	3.6	3 M KOH	2,215 F/g (1 A/g)	98.3% (7,500)	[S12]
11	$NiCo_2O_4$	0.5	3 M KOH	2,065 F/g (1 A/g)	89.3% (10,000)	[S13]
12	$Ni_{1.43}Co_{0.5}Fe_{0.5}S$	2.25	6 M KOH	1,156 C/g (1 A/g)	92.9% (10,000)	[S14]
13	Zn-Co-S	2.4-2.6	6 M KOH	971 C/g (1 A/g)	90% (10,000)	[S15]
14	Co_3O_4/NiO	1.8	2 M KOH	2,324 F/g (2 A/g)	95.5% (12,000)	[S16]
15	Cu/p-CuO/NiCo-P	1.4	2 M KOH	3,537 F/g (2 A/g)	92% (10,000)	[S17]
16	$ZnCo_{1.5}(OH)_{4.5}Cl_{0.5}\cdot 0.45H_2O$	1.1	1 M KOH	3,946.5 F/g (1 A/g)	81% (5,000)	[S18]
17	ZnNiCo-P	1.25	6 M KOH	958 C/g (1 A/g)	90% (6,000)	[S19]
18	Co_3O_4@CoNi-LDH	4.5	2 M KOH	2676.9 F/g (0.5 A/g)	67.7% (10,000)	[S20]
19	NiCo-LDH/$NiMoS_x$	3.3 ± 0.1	3 M KOH	404 mAh/g (3 mA/cm^2)	93.2% (10,000)	[S21]
20	Mn_3O_4/MnOOH	10	1 M LiCl	192.2 C/g (1 A/g)	96.7% (10,000)	[S22]
21	TGO/$Ni(OH)_2$	12.5	6 M KOH	996.2 F/g (1 A/g)	91.2% (10,00)	[S23]
22	NiCoS	10	6 M KOH	1620 F/g (1 A/g)	84% (30,00)	[S24]
23	CNT/MnO_2/GCC	9.1	1 M Na_2SO_4	371.4 F/g (1 mA/cm^2)	81.4% (10,000)	[S25]

Table S2 Fitted data of electrochemical impedance spectra shown in Fig. 4h and Fig. 5h

Electrode	R_s (Ω)	C_{dl} (mF/cm^2)	R_{CT} (Ω)	W
NCS	1.401	0.046	128.3	3.866
NCS/NMS	1.339	0.030	42.50	113.10
NCS/NMS/CoO (100 cycles)	1.312	1.216	10.21	56.12
NCS/NMS/CoO (200 cycles)	1.302	1.498	7.06	39.11
NCS/NMS/CoO (300 cycles)	1.186	1.380	14.21	35.21
NMO	1.139	0.00024	1.123	303.87
NMS	1.412	0.00134	0.456	544.62
NMS/FeO	1.478	0.02713	0.200	75.24

Table S3 Comparison of electrochemical performances of recently reported transition metal-based negative electrodes

No.	Material	Mass loading (mg/cm^2)	Electrolyte	Specific capacitance, F/g, or Capacity, C/g (Current density, A/g)	Stability (Cycles)	Refs.
1	$NiMo_2S_4/Fe_2O_3$	5	2 M KOH	1921.8 F/g or 2114 C/g (2 A/g)	93.8% (20,000)	This work
2	Fe_3O_4/ALD-TiO_2	6.3	2 M KOH	372.6 C/g (3 A/g)	90.4% (20,000)	[S11]
3	B-rGO/ALD-Fe_2O_3	5.8	2 M KOH	341.0 F/g (4 A/g)	91.0% (20,000)	[S10]
4	Fe_3O_4/FeS_2	6	1 M KOH	597.1 F/g (3 A/g)	62.1% (10,000)	[S26]
5	α-MnO_2	2	1 M KOH	736.3 F/g (1 A/g)	76.4% (10,000)	[S27]
6	Fe_3O_4/CNTs/MnO_2	-	3 M KOH	643.8 F/g (1 A/g)	-	[S28]
7	Fe_3O_4/C/CuO/CF	12	6 M KOH	1,250 F/g (5 mA/cm^2)	88.0% (5,000)	[S29]
8	Ni/CoFe-LDH	1.6	6 M KOH	1,289 F/g (1 A/g)	76.6% (1,000)	[S30]
9	Graphene/Fe_2O_3	1	1 M KOH	908 F/g (2 A/g)	75.0% (200)	[S31]
10	Graphene/Fe_2O_3	-	1 M Na_2SO_4	504 (2 mA/cm^2)	-	[S32]
11	Cu_2O-$Cu(OH)_2$-graphene	0.146	0.5 M Na_2SO_4	425 F/g (5 A/g)	87.0% (2,000)	[S33]
13	Graphene/polypyrrole/Cu_2O-$Cu(OH)_2$	1	0.5 M Na_2SO_4	997 F/g (10 A/g)	-	[S34]
14	3D MoS_2	0.5	6 M KOH	395 F/g (1.5 A/g)	86.0% (1,000)	[S35]
15	Nitrogen-doped graphene encapsulated FeS	3.2	2 M KOH	467 F/g (1 A/g)	-	[S36]

16	CuWS	0.7	1 M $LiSO_4$	626.7 F/g (5 mV/s)	-	[S37]
17	$Cu_{1.4}S$	1	-	485 F/g or 727.5 C/g (0.25 A/g)	80.2% (1,000)	[S38]
18	Graphene nanosheet/$Co_{0.33}Fe_{0.67}S_2$	2.5	3 M KOH	262 C/g (2 mV/s)	-	[S39]
19	PPy-rGO-MoS_2	2	6 M KOH	462 F/g (1 A/g)	60% (2,000)	[S40]
20	MoO_2/MoS_2	4	1 M Na_2SO_4	341.0 F/g (10 mV/s)	84.41% (5,000)	[S41]
21	MoS_2/MWCNT	-	0.5 M Na_2SO_4	266.9 F/g (0.5 A/g)	-	[S42]
22	Bi_2S_3	-	1 M KOH	532 C/g (1 A/g)	91% (3,000)	[S43]
23	rGO/Bi_2S_3	2	2 M KOH	1,932 F/g (1 A/g)	100% (700)	[S44]
24	SnS_2	5.21	1 M KCl	524.5 F/g (0.08 A/g)	-	[S45]

Table S4 Data on the thickness of the ALD coating layer for different numbers of ALD cycles

	Number of ALD cycles	Thickness (nm)
NCS/NMS/CoO	100	1.62
	200	3.24
	300	5.00
NMS/FeO	150	3.40
	300	6.80
	450	10.20

Table S5 Comparison of electrochemical performances of recently reported transition metal-based supercapacitor/supercapattery devices

No.	Supercapacitor/ Supercapattery devices	Cell voltage (V)	Electrolyte	C_s (F/g) (Current density, A/g)	Stability (Cycles)	Energy density (Wh/kg)	Power density (W/kg)	Refs.
1	$NiCo_2S_4/NiMo_2S_4/Co_3O_4$‖$NiMo_2S_4/Fe_2O_3$	1.6	2 M KOH	664.14 F/g (1062.62 C/g) (2 A/g)	75.45% (25,000)	921.94	2417.94	This work
2	Co_3O_4@CoNi-LDH//AC	1.5	2 M KOH	195.9 F/g (1 A/g)	103.5% (5,000)	61.2	750	[S20]
3	Fe_3O_4/CNTs@MnO_2// Fe_3O_4/CNTs@MnO_2	1.6	3 M KOH	195.9 F/g (1 A/g)	63% (10,000)	52.98	849.9	[S28]
4	Ni-Co-hydroxides/$Cu(OH)_2$/CF// Fe_3O_4@ carbon/CuO/CF	1.6	-	271.4 F/g (5 mA/cm^2)	86% (5,000)	90.6	188.4	[S29]
5	Co_8FeS_8@NG//FeS@NG	1.6	2 M KOH	198 F/g (1 A/g)	93.7% (10,000)	70.4	598	[S36]
6	NiCo-LDH/$NiMoS_x$//Fe_2O_3/rGO	1.6	3 M KOH	203.9 F/g	91.5 %	72.6	522.7	[S21]

				(3 mA/cm^2)	(10,000)			
7	GCNAS//GCFS-0.33	1.6	3 M KOH	187.8 F/g (2 mV/s)	~102.2% (10,000)	66.8	300.5	[S39]
8	CNTs@NCDHNs//rGO-Fe_2O_3	1.6	6 M KOH	108.7 F/g (1 A/g)	93.5% (1,000)	54.6	1130	[S46]
9	$CuCo_2O_4$@$MnMoO_4$//graphene	1.6	6 M KOH	165.7 F/g (1 A/g)	92.5% (6,000)	58.9	670	[S47]
10	GA@UiO-66-NH_2//$Ti_3C_2T_x$	1.6	1 M Na_2SO_4	212 F/g (1.3 A/g)	88% (10,000)	73	1,000	[S48]
11	$NiFe_2O_4$-NP-NS@CC//NPC	1.6	6 M KOH	202 F/g (0.5 A/g)	94.2 % (5,000)	69	771	[S49]
12	CuS@Cu_2O//Bi_2O_3	1.6	0.1 M KOH	559 F/g (0.8 A/g)	87.23% (5,000)	52	750	[S50]
13	$Zn_xCo_{1-x}O$//$Zn_xCo_{1-x}O$	1.5	6 M KOH	450 F/g (1 V/s)	90.7% (5,000)	67.3	1,670	[S51]
14	$NiCo_2S_4$@NC-array//RGO@NF	1.6	3 M KOH	23.4 mAh/g (0.5 A/g)	93.9% (3,000)	66.5	400	[S52]
15	Cu/p-CuO/NiCo–P//3DPG	1.6	2 M KOH	250 F/g(1 A/g)	89% (10,000)	88.1	800.6	[S17]
16	$ZnCo_{1.5}(OH)_{4.5}Cl_{0.5}\cdot 0.45H_2O$//rGO	1.5	1 M KOH	255.8 F/g (1 A/g)	81% (5,000)	114.8	643.8	[S18]
17	$NiCo_2O_4$/MoO_2@ALD-NiO//B-RGO@ALD-Fe_2O_3	1.8	2 M KOH	545.7 C/g (2 A/g)	92.3% (20,000)	136	1,800	[S10]
18	rGO/$CuCo_2O_4$/Ni_4Mo/MoO_2/ALD-Co_3O_4//Fe_3O_4/ALD-TiO_2	1.7	2 M KOH	467.6 C/g (3 A/g)	90% (20,000)	110.4	2,184	[S11]
19	$CoSnO_3$@RGO//B-RGO	1.5	2 M KOH	286.1 C/g (2 A/g)	93.1% (10,000)	76.1	1,915.8	[S53]
20	5 nm NiO/Co_3O_4@NF//AC	1.7	2 M KOH	358.7 C/g (5 A/g)	95.5% (12,000)	81.45	4,268	[S16]

Table S6 Fitted data of electrochemical impedance spectra shown in Fig. S24

	NCS/NMS/CoO‖NMS/FeO	NCS/NMS/CoO‖AC
R_s (Ω)	4.57	4.14
R_{bulk} (Ω)	1.58	2.52
C_{bulk} (mF/cm^2)	0.0002034	0.0002609
R_{CT} (Ω)	7.31	17.54
C_{dl} (mF/cm^2)	0.029757	0.51024
W	58.12	-

Supplementary References

[S1] S. Seenivasan, H. Jung, J.W. Han, D.H. Kim, Surface roughening strategy for highly efficient bifunctional electrocatalyst: combination of atomic layer deposition and anion exchange reaction. Small Methods **6**(2), 2101308 (2022). https://doi.org/10.1002/smtd.202101308

[S2] F. Wang, X. Wang, Z. Chang, Y. Zhu, L. Fu et al., Electrode materials with tailored facets for electrochemical energy storage. Nanoscale Horizons. **1**(4), 272-289 (2016). https://doi.org/10.1039/C5NH00116A

[S3] A. Kiejna, T. Pabisiak, Mixed termination of hematite (α-Fe_2O_3)(0001) surface. J. Phys. Chem. C **117**(46), 24339-24344 (2013). https://doi.org/10.1021/jp406946s

[S4] J.F. Yu, L.Y. Lin. Structure variation of nickel cobalt sulfides using Ni foam and nickel salt as the nickel source and the application on the supercapacitor electrode. J. Energy Storage **7**, 295-304 (2016). https://doi.org/10.1016/j.est.2016.08.004

[S5] K.J. Huang, J.Z. Zhang, Y. Liu, Y.M. Liu, Synthesis of reduced graphene oxide wrapped-copper sulfide hollow spheres as electrode material for supercapacitor. Int. J. Hydrogen Energy **40**(32), 10158-10167 (2015). https://doi.org/10.1016/j.ijhydene.2015.05.152

[S6] C.C. Tu, L.Y. Lin, B.C. Xiao, Y.S. Chen, Highly efficient supercapacitor electrode with two-dimensional tungsten disulfide and reduced graphene oxide hybrid nanosheets. J. Power Sources **320**, 78-85 (2016). https://doi.org/10.1016/j.jpowsour.2016.04.083

[S7] P. Naveenkumar, G.P. Kalaignan, Electrodeposited MnS on graphene wrapped Ni-foam for enhanced supercapacitor applications. Electrochim. Acta **289**, 437-447 (2018). https://doi.org/10.1016/j.electacta.2018.09.100

[S8] L. Fang, Y. Qiu, T. Zhai, F. Wang, M. Lan et al., Flower-like nanoarchitecture assembled from Bi_2S_3 nanorod/MoS_2 nanosheet heterostructures for high-performance supercapacitor electrodes. Colloids Surf. A Physicochem. Eng. Asp. **535**, 41-48 (2017). https://doi.org/10.1016/j.colsurfa.2017.09.022

[S9] Q. Pan, Y. Liu, L. Zhao, Co_9S_8/Mo_2S_3 nanorods on CoS_2 laminar arrays as advanced electrode with superior rate properties and long cycle life for asymmetric supercapacitors. Chem. Eng. J. **351**, 603-612 (2018). https://doi.org/10.1016/j.cej.2018.06.131

[S10] T. Kavinkumar, S. Seenivasan, H.H. Lee, H. Jung, J.W. Han et al., Interface-modulated uniform outer nanolayer: a category of electrodes of nanolayer-encapsulated core-shell configuration for supercapacitors. Nano Energy **81**, 105667 (2020). https://doi.org/10.1016/j.nanoen.2020.105667

[S11] T. Kavinkumar, S. Seenivasan, A.T. Sivagurunathan, Y. Kwon, D.H. Kim, Three-dimensional hierarchical core/shell electrodes using highly conformal TiO_2 and Co_3O_4 thin films for high-performance supercapattery devices. ACS Appl. Mater. Interfaces **13**(24), 29058–29069 (2021). https://doi.org/10.1021/acsami.1c04572

[S12] G. Li, B. Song, X. Cui, H. Ouyang, K. Wang et al., Multidimensional and binary micro $CuCo_2O_4$/nano $NiMoO_4$ for high-performance supercapacitors. ACS Sustain. Chem. Eng. **8**(3), 1687-1694 (2020). https://doi.org/10.1021/acssuschemeng.9b07356

[S13] S. Pappu, K. Nanaji, S. Mandati, T.N. Rao, S.K. Martha et al., Cost-effective synthesis of electrodeposited $NiCo_2O_4$ nanosheets with induced oxygen vacancies: a highly efficient electrode material for hybrid supercapacitors. Batteries Supercaps **3**(11), 1209-1219 (2020). https://doi.org/10.1002/batt.202000121

[S14] K. Alitabar, A.M. Zardkhoshoui, S.S.H. Davarani, One-step synthesis of porous

Ni−Co−Fe−S nanosheet arrays as an efficient battery-type electrode material for hybrid supercapacitors. Batteries Supercaps **3**(12), 1311-1320 (2020). https://doi.org/10.1002/batt.202000141

[S15] J. Zhao, S. Hou, Y. Bai, Y. Lian, Q. Zhou et al., Multilayer dodecahedrons Zn-Co sulfide for supercapacitors. Electrochim. Acta **354**, 136714 (2020). https://doi.org/10.1016/j.electacta.2020.136714

[S16] S. Adhikari, S. Selvaraj, S.H. Ji, D.H. Kim, Encapsulation of Co_3O_4 nanocone arrays via ultrathin NiO for superior performance asymmetric supercapacitors. Small **16**(48), 2005414 (2020). https://doi.org/10.1002/smll.202005414

[S17] J. Huang, Y. Xiong, Z. Peng, L. Chen, L. Wang et al., A general electrodeposition strategy for fabricating ultrathin nickel cobalt phosphate nanosheets with ultrahigh capacity and rate performance. ACS Nano **14**(10), 14201-14211 (2020). https://doi.org/10.1021/acsnano.0c07326

[S18] Z. Pan, Y. Jiang, P. Yang, Z. Wu, W. Tian et al., In situ growth of layered bimetallic ZnCo hydroxide nanosheets for high-performance all-solid-state pseudocapacitor. ACS Nano **12**(3), 2968-2979 (2018). https://doi.org/10.1021/acsnano.8b00653

[S19] J. Li, Z. Liu, Q. Zhang, Y. Cheng, B. Zhao et al., Anion and cation substitution in transition-metal oxides nanosheets for high-performance hybrid supercapacitors. Nano Energy **57**, 22-33 (2019). https://doi.org/10.1016/j.nanoen.2018.12.011

[S20] J.J. Zhou, Q. Li, C. Chen, Y.L. Li, K. Tao et al., Co_3O_4@CoNi-LDH core/shell nanosheet arrays for high-performance battery-type supercapacitors. Chem. Eng. J. **350**, 551-558 (2018). https://doi.org/10.1016/j.cej.2018.06.023

[S21] S. Kandula, K.R. Shrestha, G. Rajeshkhanna, N.H. Kim, J.H. Lee, Kirkendall growth and ostwald ripening induced hierarchical morphology of Ni–Co LDH/$MMoS_x$ (M = Co, Ni, and Zn) heteronanostructures as advanced electrode materials for asymmetric solid-state supercapacitors. ACS Appl. Mater. Interfaces **11**(12), 11555-11567 (2019). https://doi.org/10.1021/acsami.9b02978

[S22] W. Zheng, J. Halim, Z. Sun, J. Rosen, M.W. Barsoum, MXene—manganese oxides aqueous asymmetric supercapacitors with high mass loadings, high cell voltages and slow self-discharge. Energy Storage Mater. **38**, 438-446 (2021). https://doi.org/10.1016/j.ensm.2021.03.011

[S23] Y. Tang, Y. Liu, W. Guo, T. Chen, H. Wang et al., Highly oxidized graphene anchored $Ni(OH)_2$ nanoflakes as pseudocapacitor materials for ultrahigh loading electrode with high areal specific capacitance. J. Phys. Chem. C **118**(43), 24866-24876 (2014). https://doi.org/10.1021/jp5075779

[S24] Y. Wen, Y. Liu, T. Wang, Z. Wang, Y. Zhang et al., High-mass-loading Ni–Co–S electrodes with unfading electrochemical performance for supercapacitors. ACS Appl. Energy Mater. **4**(7), 6531-6541 (2021). https://doi.org/10.1021/acsaem.1c00557

[S25] L. Lyu, K. Seong, J.M. Kim, W. Zhang, X. Jin et al., CNT/high mass loading MnO_2/graphene-grafted carbon cloth electrodes for high-energy asymmetric supercapacitors. Nano-Micro Lett. **11**, 88 (2019). https://doi.org/10.1007/s40820-019-0316-7

[S26] X. Liu, L. Liu, W. Yan, Y. Wang, C. Huang et al., Hierarchical Fe_3O_4@FeS_2 nanocomposite as high-specific-capacitance electrode material for supercapacitors. Energy Technol. **8**(10), 2000544 (2020). https://doi.org/10.1002/ente.202000544

[S27] Y. Chen, C. Zhou, G. Liu, C. Kang, L. Ma et al., Hydroxide ion dependent α-MnO_2 enhanced via oxygen vacancies as the negative electrode for high-performance supercapacitors. J. Mater. Chem. **9**(5), 2872-2887 (2021). https://doi.org/10.1039/D0TA10489B

[S28] N. Wang, G. Ding, X. Yang, L. Zhao, D. He, Membrane MnO_2 coated Fe_3O_4/CNTs negative material for efficient full-pseudocapacitance supercapacitor. Mater. Lett. **255**, 126589 (2019). https://doi.org/10.1016/j.matlet.2019.126589

[S29] D. Zhang, Y. Shao, X. Kong, M. Jiang, X. Lei, Hierarchical carbon-decorated Fe_3O_4 on hollow CuO nanotube array: fabrication and used as negative material for ultrahigh-energy density hybrid supercapacitor. Chem. Eng. J. **349**, 491-499 (2018). https://doi.org/10.1016/j.cej.2018.05.120

[S30] S. Verma, V. Gupta, A. Khosla, S. Kumar, S. Arya, High performance asymmetric supercapacitor based on vertical nanowire arrays of a novel Ni@Co–Fe LDH core@shell as negative and $Ni(OH)_2$ as positive electrode. Nanotechnology **31**(24), 245401 (2020). https://doi.org/10.1088/1361-6528/ab7b07

[S31] H. Wang, Z. Xu, H. Yi, H. Wei, Z. Guo et al., One-step preparation of single-crystalline Fe_2O_3 particles/graphene composite hydrogels as high performance anode materials for supercapacitors. Nano Energy **7**, 86-96 (2014). https://doi.org/10.1016/j.nanoen.2014.04.009

[S32] Q.X. Low, G.W. Ho, Facile structural tuning and compositing of iron oxide-graphene anode towards enhanced supacapacitive performance. Nano Energy **5**, 28-35 (2014). https://doi.org/10.1016/j.nanoen.2014.01.002

[S33] S. Ghasemi, M. Jafari, F. Ahmadi, Cu_2O-$Cu(OH)_2$-graphene nanohybrid as new capacitive material for high performance supercapacitor. Electrochim. Acta **210**, 225-235 (2016). https://doi.org/10.1016/j.electacta.2016.05.155

[S34] P. Asen, S. Shahrokhian, A high performance supercapacitor based on graphene/polypyrrole/Cu_2O–$Cu(OH)_2$ ternary nanocomposite coated on nickel foam. J. Phys. Chem. C **121**(12), 6508-6519 (2017). https://doi.org/10.1021/acs.jpcc.7b00534

[S35] A.M. Abraham, S.P. Lonkar, V.V. Pillai, S.M. Alhassan, Three-dimensional MoS_2 nanodot-impregnated nickel foam electrodes for high-performance supercapacitor applications. ACS Omega **5**(20), 11721-11729 (2020). https://doi.org/10.1021/acsomega.0c01045

[S36] Y.A. Haj, J. Balamurugan, N.H. Kim, J.H. Lee, Nitrogen-doped graphene encapsulated cobalt iron sulfide as an advanced electrode for high-performance asymmetric supercapacitors. J. Mater. Chem. **7**(8), 3941-3952 (2019). https://doi.org/10.1039/C8TA12396A

[S37] P. Pazhamalai, K. Krishnamoorthy, S. Sahoo, V.K. Mariappan, S.J. Kim, Copper tungsten sulfide anchored on Ni-foam as a high-performance binder free negative electrode for asymmetric supercapacitor. Chem. Eng. J. **359**, 409-418 (2019). https://doi.org/10.1016/j.cej.2018.11.153

[S38] X. He, X. Mao, C. Zhang, W. Yang, Y. Zhou et al., Flexible binder-free hierarchical copper sulfide/carbon cloth hybrid supercapacitor electrodes and the application as negative electrodes in asymmetric supercapacitor. J. Mater. Sci. Mater. Electron. **31**(3), 2145-2152 (2020). https://doi.org/10.1007/s10854-019-02737-4

[S39] W. Liu, H. Niu, J. Yang, K. Cheng, K. Ye et al., Ternary transition metal sulfides embedded in graphene nanosheets as both the anode and cathode for high-performance asymmetric supercapacitors. Chem. Mater. **30**(3), 1055-1068 (2018). https://doi.org/10.1021/acs.chemmater.7b04976

[S40] Y.X. Chen, W.J. Ma, K.F. Cai, X.W. Yang, C.J. Huang, In situ growth of polypyrrole onto three-dimensional tubular MoS_2 as an advanced negative electrode material for supercapacitor. Electrochim. Acta **246**, 615-624 (2017). https://doi.org/10.1016/j.electacta.2017.06.102

[S41] T. Zhang, L.B. Kong, M.C. Liu, Y.H. Dai, K. Yan et al., Design and preparation of MoO_2/MoS_2 as negative electrode materials for supercapacitors. Mater. Design **112**, 88-96 (2016). https://doi.org/10.1016/j.matdes.2016.09.054

[S42] B. Cheng, R. Cheng, F. Tan, X. Liu, J. Huo et al., Highly efficient quasi-solid-state asymmetric supercapacitors based on MoS_2/MWCNT and PANI/MWCNT composite electrodes. Nanoscale Res. Lett. **14**(1), 66 (2019). https://doi.org/10.1186/s11671-019-2902-5

[S43] X. Yu, J. Zhou, Q. Li, W.N. Zhao, S. Zhao et al., Bi_2S_3 nanorod-stacked hollow microtubes self-assembled from bismuth-based metal–organic frameworks as advanced negative electrodes for hybrid supercapacitors. Dalton Trans. **48**(25), 9057-9061 (2019). https://doi.org/10.1039/C9DT01466G

[S44] K. Ghosh, S.K. Srivastava, Superior supercapacitor performance of Bi_2S_3 nanorod/reduced graphene oxide composites. Dalton Trans. **49**(46), 16993-17004 (2020). https://doi.org/10.1039/D0DT03594G

[S45] R.K. Mishra, G.W. Baek, K. Kim, H.I. Kwon, S.H. Jin, One-step solvothermal synthesis of carnation flower-like SnS_2 as superior electrodes for supercapacitor applications. Appl. Surf. Sci. **425**, 923-931 (2017). https://doi.org/10.1016/j.apsusc.2017.07.045

[S46] Y. Wang, H. Wei, H. Lv, Z. Chen, J. Zhang et al., Highly stable three-dimensional nickel–cobalt hydroxide hierarchical heterostructures hybridized with carbon nanotubes for high-performance energy storage devices. ACS Nano **13**(10), 11235-11248 (2019). https://doi.org/10.1021/acsnano.9b04282

[S47] S. Chen, S. Cui, S. Chandrasekaran, C. Ke, Z. Li et al., Growth of $CuCo_2O_4$@$MnMoO_4$ core/shell nanosheet arrays for high energy density asymmetric supercapacitors. Electrochim. Acta **341**, 135893 (2020). https://doi.org/10.1016/j.electacta.2020.135893

[S48] K. Jayaramulu, M. Horn, A. Schneemann, H. Saini, A. Bakandritsos et al., Covalent graphene-MOF hybrids for high-performance asymmetric supercapacitors. Adv. Mater. **33**(4), 2004560 (2021). https://doi.org/10.1002/adma.202004560

[S49] J. Xi, M.S. Javed, S. Asim, M. Idrees, S.S.A. Shah et al., High-performance flexible supercapatteries enabled by binder-free two-dimensional mesoporous ultrathin nickel-ferrite nanosheets. Mater. Chem. Front. **5**(8), 3436-3447 (2021). https://doi.org/10.1039/D1QM00109D

[S50] H.W. Lee, N.M. Shinde, P.V. Shinde, J.M. Yun, P.K. Song et al., High energy and power density of self-grown CuS@Cu_2O core-shell supercapattery positrode. J. Solid State Electrochem. **23**(9), 2609-2617 (2019). https://doi.org/10.1007/s10008-019-04351-0

[S51] T. Ling, P. Da, X. Zheng, B. Ge, Z. Hu et al., Atomic-level structure engineering of metal oxides for high-rate oxygen intercalation pseudocapacitance. Sci. Adv. **4**(10), eaau6261 (2018). https://doi.org/10.1126/sciadv.aau6261

[S52] Z. Hao, X. He, H. Li, D. Trefilov, Y. Song et al., Vertically aligned and ordered arrays of 2D MCo_2S_4@metal with ultrafast ion/electron transport for thickness-independent pseudocapacitive energy storage. ACS Nano **14**(10), 12719-12731 (2020). https://doi.org/10.1021/acsnano.0c02973

[S53] T. Kavinkumar, H.H. Lee, D.H. Kim, Design of all-solid-state hybrid supercapacitor based on mesoporous $CoSnO_3$@RGO nanorods and B-doped RGO nanosheets grown on Ni foam for energy storage devices of high energy density. Appl. Surf. Sci. **541**, 148354 (2021). https://doi.org/10.1016/j.apsusc.2020.148354
